# Supplementary material for: Genetic Determinants of Plasma Low-Density Lipoprotein Cholesterol Levels: Monogenicity, Polygenicity, and “Missing” Heritability
Source: Biomedicines. 2021 Nov 19;9(11):1728. doi: 10.3390/biomedicines9111728 (PMC8615680; doi:10.3390/biomedicines9111728)
Supplement: Supplementary file 1 [file biomedicines-09-01728-s001.zip › biomedicines-1435333-sup2.pdf]

Supplementary material for **Genetic Determinants of Plasma Low-Density Lipoprotein Cholesterol Levels: Monogenicity, Polygenicity, and "Missing" Heritability.**

Supplementary methods

Search for GWASs related to plasma LDL-c levels: The GWAS Catalog (www.ebi.ac.uk/gwas) was accessed on August 18, 2021. After a search for the trait "low-density lipoprotein cholesterol measurement" (EFO_0004611), two .csv files were downloaded: a file with the published studies included in the database (study file) and a file with the SNVs associated with the trait in these studies (association file). The first step was to filter the "reported trait" field of the study file using "Low-density lipoprotein cholesterol levels", "LDL cholesterol", "LDL cholesterol levels", "Total cholesterol levels in LDL", "Lipid traits", "Cardiovascular disease risk factors", "Metabolite levels", "Cholesterol", "Lipid or lipoprotein levels", "Quantitative traits", "Biochemical measures", or "Cardiovascular risk factors." The next step was to choose discovery samples from the following groups: Europeans or European ancestry, East Asians, Afro-Americans or African ancestry, Native Americans or Hispanics, Middle-East populations, and Oceanians. Studies of multiethnic discovery samples were discarded. Studies with consigned background traits (usually a disease) were also discarded. The selected studies were used to extract SNVs associated with LDL-c in the association file. All the SNV were checked through the dbSNP (www.ncbi.nlm.nih.gov/snp) and the ClinVar (www.ncbi.nlm.nih.gov/clinvar) databases to determine the type of mutation. Polyphen (genetics.bwh.harvard.edu/pph2/) and Sift (sift.bii.a-star.edu.sg/) programs were used for the prediction of the functional effects of SNVs affecting exons.

Table S1a: SNVs associated with low-density lipoprotein cholesterol in European ancestry populations.

Mapped gene[a]	Region	SNPid[b]	Chr	Position[c]	LD[d]	Type[e]	cDNA	Protein	ClinVar[f]	Polyphen[g]	Sift[h]	p-value	References
SKI		rs560221037	1	2,235,046		intronic						6E−12	[22]
TTC34 - ACTRT2		rs79316815	1	2,902,742		intergenic						3E−7	[17]
CASZ1		rs880315	1	10,736,809		intronic						7E−12	[21]
CASZ1		rs682178	1	10,738,495		intronic						5E−10	[22]
CLCN6		rs112521149	1	11,829,758		intronic						4E−8	[22]
EPHA2 - ARHGEF19-AS1		rs12078100	1	16,186,091		intergenic						7E−13; 9E−10; 2E−24	[20][21][22]
ASAP3		rs61778883	1	23,468,684		intronic						2E−9	[21]
ASAP3		rs375390883	1	23,468,691		intronic						3E−17	[22]
ELOA		chr1:23747996	1	23,747,996		intergenic						4E−9	[20]
			1	25,420,739		missense	c.48G>C	p.Trp16Cys	no interp.	benign	tolerated	4E−9	[13]
			1	25,442,719		intronic						4E−36	[20]
			1	25,449,242		intronic						1E−10; 2E−14	[8][12]
			1	25,467,172		intronic						2E−35	[21]
ARID1A		rs114165349	1	26,695,422		intronic						4E−27; 8E−16	[20][21]
PIGV - RN7SL165P		rs12748152	1	26,811,902		intergenic						3E−12	[12]
KDF1		rs79598313	1	26,958,422		intronic						7E−10	[13]
MECR		rs2291609	1	29,224,479		intronic						2E−8	[20]
			1	54,646,717		intergenic						1E−7	[15]
			1	54,876,728		intronic						2E−9	[15]
			1	54,983,266		intronic						3E−9	[15]
			1	55,030,366		intergenic						4E−11; 4E−8; 1E−10	[1][4][9]
			1	55,038,977		5'-UTR	c.-861G>A		benign			2E−28; 3E−50	[8][12]

Mapped gene[a]	Region	SNPid[b]	Chr	Position[c]	LD[d]	Type[e]	cDNA	Protein	ClinVar[f]	Polyphen[g]	Sift[h]	p-value	References
PCSK9	*PCSK9*	rs11591147	1	55,039,974		missense	c.137G>T	p.Arg46Leu	benign/likely benign	benign	tolerated	2E−44; 2E−92; 3E−563; 2E−421	[2][13][20][21]
PCSK9		rs693668	1	55,055,436		intronic						9E−8	[19]
PCSK9		rs472495	1	55,055,640		intronic						6E−85	[21]
PCSK9		rs11206517	1	55,060,755		intronic						6E−32	[21]
USP24 - Y_RNA		chr1:55246601	1	55,246,601		intergenic						4E−16	[15]
USP24 - Y_RNA		chr1:55505647	1	55,505,647		intergenic						1E−29	[15]
USP24 - Y_RNA		chr1:55636240	1	55,636,240		intergenic						6E−25	[15]
USP24 - Y_RNA		chr1:55759138	1	55,759,138		intergenic						8E−21	[15]
USP24 - Y_RNA		chr1:55939497	1	55,939,497		intergenic						2E−18	[15]
USP24 - Y_RNA		chr1:56062375	1	56,062,375		intergenic						3E−17	[15]
ENSG00000284686		rs17457613	1	56,250,181		intronic						2E−9	[20]
ENSG00000284686		chr1:56304721	1	56,304,721		intronic						2E−15	[15]
ENSG00000284686		rs11206788	1	56,332,309		intronic						2E−10	[21]
ENSG00000284686		chr1:56480207	1	56,480,207		intronic						2E−15	[15]
FYB2 - C8A		chr1:56840574	1	56,840,574		intergenic						3E−14	[15]
DAB1		chr1:56995693	1	56,995,693		3'-UTR	c.*2451C>?					5E−9	[15]
DOCK7	*DOCK7 - ANGPTL3*	rs2131925	1	62,560,271		intronic						3E−18; 3E−32	[8][12]
DOCK7		rs10889348	1	62,612,551		intronic						2E−11	[13]
DOCK7		rs10889353	1	62,652,525		intronic						8E−6	[5]
DOCK7		rs1168127	1	62,670,407		intronic						2E−70	[21]
ATG4C - FOXD3		chr1:62900811	1	62,900,811		intergenic						2E−6	[15]
ATG4C - FOXD3		chr1:62944947	1	62,944,947		intergenic						5E−101	[20]
ATG4C - FOXD3		chr1:63086001	1	63,086,001		intergenic						4E−6	[15]
GFI1 - EVI5		rs11164654	1	92,502,755		intergenic						2E−8	[13]
EVI5		rs1556562	1	92,568,466		intronic						2E−12; 2E−14	[20][21]
Y_RNA - PRMT6		rs3108680	1	107,030,375		intergenic						2E−9	[20]

Mapped gene[a]	Region	SNPid[b]	Chr	Position[c]	LD[d]	Type[e]	cDNA	Protein	ClinVar[f]	Polyphen[g]	Sift[h]	p-value	References
			1	108,925,836		intronic						6E−10	[21]
			1	109,233,663		intronic						8E−304	[22]
			1	109,255,141		intronic						3E−40	[21]
			1	109,264,661		intronic						1E−277	[20]
			1	109,272,258		intronic						2E−336	[21]
			1	109,274,968		3'-UTR	c.*919G>T		association			2E−42	[4]
			1	109,275,216		3'-UTR	c.*1167T>C		np			1E−26	[9]
			1	109,275,684		3'-UTR	c.*1635G>C		np			1E−170; 5E−241; 2E−72	[8][12][19]
			1	109,275,908		intergenic						3E−29; 8E−23; 2E−12; 2E−91	[2][5][6][13]
			1	109,279,544		intergenic						6E−33; 1E−33	[1][3]
			1	109,519,352		intergenic						7E−11	[21]
			1	109,690,361		missense	c.451A>G	p.Asn151Asp	np	benign	tolerated	6E−10	[21]
			1	109,817,590		intergenic						4E−15	[15]
CIART		rs2147324	1	150,283,177		intronic						3E−8	[20]
TARS2		rs77257036	1	150,503,710		intronic						4E−8	[21]
ANXA9		rs267733	1	150,986,360		missense	c.497A>G	p.Asp166Gly	np	probably damaging	tolerated	5E−9	[12]
Y_RNA - EFNA1		rs11807418	1	155,122,129		intergenic						4E−8	[20]
EFNA1		rs4745	1	155,133,751		missense	c.476A>T	p.Asp159Val	np	benign	tolerated	3E−10	[16]
LAMC21 - LAMC2		rs35221132	1	183,145,730		intergenic						8E−9	[20]
PTPRC - LINC01221		chr1:198994696	1	198,994,696		intergenic						2E−9	[20]
LINC01221		rs6667939	1	199,025,490		intergenic						7E−11	[21]
CR1L		rs4844614	1	207,701,830		intronic						2E−7	[6]
			1	220,796,686		missense	c.493A>G	p.Thr165Ala	np	benign	tolerated	3E−33; 8E−29	[20][21]

Mapped gene[a]	Region	SNPid[b]	Chr	Position[c]	LD[d]	Type[e]	cDNA	Protein	ClinVar[f]	Polyphen[g]	Sift[h]	p-value	References
MTARC1	*MTARC1*	rs2642442	1	220,800,221		intronic						6E−11; 5E−11	[8][12]
CNIH4, NVL		rs12751807	1	224,349,953		intergenic						1E−9	[20]
IRF2BP2 - TOMM20	*IRF2BP2*	rs10910476	1	234,599,210		intergenic						4E−9	[21]
IRF2BP2 - TOMM20		rs556107	1	234,717,312		intergenic						1E−70; 8E−64	[20][21]
IRF2BP2 - TOMM20		rs514230	1	234,722,850		intergenic						9E−12; 9E−12; 1E−11	[8][12][13]
IRF2BP2 - TOMM20		rs28631087	1	234,973,467		intergenic						2E−10	[21]
RPS7, COLEC11		rs56236159	2	3,588,888		intergenic						7E−9	[21]
COLEC11, RPS7		rs6542680	2	3,592,552		intergenic						1E−9	[20]
COLEC11, RPS7		rs3820897	2	3,594,771		intergenic						2E−14	[22]
LINC01814		rs72784625	2	8,580,520		intergenic						6E−9	[22]
LINC00570		rs72774870	2	11,372,699		intergenic						4E−8	[21]
RN7SL140P, RPS16P2	*APOB*	rs907866	2	20,171,619		intergenic						4E−17	[21]
LDAH		rs4971516	2	20,703,255		intronic						2E−52	[10]
APOB		rs13392272	2	20,994,618		intergenic						1E−306	[20]
APOB		rs693	2	21,009,323		synonymous	c.7545C>T	p.Thr2515=	benign			1E−21; 4E−17; 3E−11	[2][5][6]
APOB		rs1367117	2	21,041,028		missense	c.293C>T	p.Thr98Ile	benign/likely benign	benign	deleterious	4E−114; 1E−182	[8][12]
TDRD15, APOB		rs934197	2	21,044,589		intergenic						9E−312	[21]
TDRD15, APOB		rs7575840	2	21,050,618		intergenic						4E−28	[19]
APOB, TDRD15		rs515135	2	21,063,185		intergenic						5E−29; 2E−20; 2E−63	[4][9][13]
APOB, TDRD15		rs562338	2	21,065,449		intergenic						6E−22; 1E−9	[1][3]
TDRD15 - NUTF2P8		rs312976	2	21,152,474		intergenic						5E−324	[22]
TDRD15 - NUTF2P8		rs430096	2	21,211,708		intergenic						2E−103	[21]
TDRD15 - NUTF2P8		chr2:21281097	2	21,281,097		intergenic						8E−13	[15]

Mapped gene[a]	Region	SNPid[b]	Chr	Position[c]	LD[d]	Type[e]	cDNA	Protein	ClinVar[f]	Polyphen[g]	Sift[h]	p-value	References
TDRD15 - NUTF2P8	*APOB*	chr2:21383353	2	21,383,353		intergenic						4E−10	[15]
TDRD15 - NUTF2P8		rs11693526	2	22,124,628		intergenic						2E−8; 7E−6	[20][21]
ITSN2		rs77907512	2	24,267,100		intronic						5E−11	[20]
KCNK3		rs1731243	2	26,707,543		intronic						7E−10	[21]
GCKR	*GCKR*	rs1260326	2	27,508,073		missense	c.1337T>C	p.Leu446Pro	benign, association	possibly damaging	tolerated	4E−99; 6E−60	[20][21]
THADA	*ABCG5/8*	rs12990177	2	43,455,520		intronic						1E−12	[21]
ABCG5		rs6756629	2	43,837,951		missense	c.148C>T	p.Arg50Cys	benign/likely benign	probably damaging	deleterious	3E−10	[5]
ABCG8		rs4299376	2	43,845,437		intronic						2E−47; 4E−72; 5E−187; 4E−131	[8][12][20] [21]
ABCG8		rs41360247	2	43,846,517		intronic						1E−10	[19]
ABCG8		rs6544713	2	43,846,742		intronic						2E−20	[4]
ABCG8		rs72875462	2	43,852,171		intronic						2E−35	[13]
ABCG8		rs6709904	2	43,853,185		intronic						9E−40	[21]
EHBP1		rs4671050	2	62,761,034		intronic						1E−21	[20]
EHBP1		rs7562734	2	62,820,838		intronic						2E−19	[21]
EHBP1		rs2710642	2	62,922,422		intronic						6E−9	[12]
SERTAD2		rs1861398	2	64,679,666		intronic						8E−9	[20]
SERTAD2		rs12471768	2	64,701,469		intronic						3E−9	[21]
GCC2		rs763102966	2	108,449,778		intronic						1E−12	[20]
LIMS1		rs2718717	2	108,589,683		intronic						3E−10	[21]
IL1F10		rs3811055	2	113,073,605		intronic						2E−8	[20]
INSIG2, RN7SL111P	*INSIG2*	rs10490626	2	118,078,265		intergenic						2E−12	[12]
RN7SL111P, INSIG2		rs150474434	2	118,087,545		intergenic						7E−22; 1E−23	[20][21]
LINC01101		rs17050272	2	120,548,864		intergenic						2E−24; 3E−22	[20][21]
Y_RNA, LINC01101		rs2030746	2	120,551,912		intergenic						9E−9	[12]
TMEM163		rs10928512	2	134,693,732		intronic						4E−8	[19]

Mapped gene[a]	Region	SNPid[b]	Chr	Position[c]	LD[d]	Type[e]	cDNA	Protein	ClinVar[f]	Polyphen[g]	Sift[h]	p-value	References
ACMSD, CCNT2-AS1		rs4954192	2	134,875,411		intronic						6E−12	[21]
ZRANB3		rs151022760	2	135,340,991		intronic						4E−12	[20]
DARS1-AS1, CXCR4		rs6714750	2	136,025,599		intergenic						3E−8	[21]
ACVR1C		rs12614487	2	157,578,057		intronic						5E−10	[20]
ACVR1C		rs7601153	2	157,591,059		intronic						2E−7	[21]
GRB14, COBLL1		rs10184004	2	164,651,879		intergenic						1E−11	[20]
ABCB11		rs2287622	2	168,973,818		missense	c.1331T>C	p.Val444Ala	benign	benign	tolerated	3E−23	[21]
UBR3		chr2:169829810	2	169,829,810		intronic						8E−24	[20]
FAM117B		rs7569317	2	202,663,256		intronic						9E−18	[21]
CARF		rs140244541	2	202,943,809		intronic						6E−9	[13]
RAPH1		rs7603427	2	203,452,830		intronic						9E−8	[21]
RAPH1 - CD28		chr2:203556224	2	203,556,224		intergenic						1E−24	[20]
FN1		rs1250258	2	215,435,462		intronic						7E−9	[21]
FN1		rs1250229	2	215,439,661		intergenic						3E−8	[12]
UGT1A3, UGT1A5, UGT1A9, UGT1A10, UGT1A4, UGT1A6, UGT1A8, UGT1A7		rs11568318	2	233,756,852		intronic						7E−10; 7E−19	[21][22]
UGT1A8, UGT1A9, UGT1A4, UGT1A7, UGT1A10, UGT1A6, UGT1A5, UGT1A1, UGT1A3		rs11563251	2	233,770,738		intronic						5E−8; 8E−13	[12][20]
FARP2		rs59916403	2	241,431,336		intronic						4E−9	[22]
GSTM5P1, PPARG	*PPARG*	rs2920503	3	12,282,731		intergenic						3E−10	[13]
GSTM5P1, PPARG		rs13076933	3	12,285,932		intergenic						6E−26; 3E−18; 8E−49	[20][21][22]
CMTM6		rs7640978	3	32,491,518		intronic						1E−8	[12]
CMTM6		rs9834932	3	32,493,890		intronic						1E−18	[21]

Mapped gene[a]	Region	SNPid[b]	Chr	Position[c]	LD[d]	Type[e]	cDNA	Protein	ClinVar[f]	Polyphen[g]	Sift[h]	p-value	References
CMTM6		rs150605723	3	32,499,834		intronic						2E−19	[20]
PXK		rs9825431	3	58,420,967		intronic						4E−18	[20]
KCTD6, PDHB		rs71311871	3	58,434,886		intergenic						7E−14	[21]
MITF		rs55921103	3	69,761,143		intronic						3E−12; 5E−10	[20][21]
MITF		rs7623486	3	69,859,943		intronic						9E−7	[17]
NR1I2		rs3732359	3	119,817,582		3'-UTR	c.*370G>A		np			5E−12	[21]
GSK3B		rs570238285	3	120,069,666		intronic						1E−8	[20]
CASR		chr3:122285218	3	122,285,218		3'-UTR	c.*27A>?					1E−19	[20]
PARP9		rs9841897	3	122,563,722		intronic						1E−10	[21]
PLXND1		rs56299595	3	129,559,339		intronic						2E−8	[20]
DNAJC13		rs17404153	3	132,444,356		intronic						2E−9	[12]
DNAJC13		rs113177823	3	132,498,859		intronic						8E−20; 1E−18	[20][21]
STAG1		rs28478252	3	136,402,758		intronic						4E−11	[20]
STAG1		rs3932048	3	136,540,082		intronic						6E−10	[21]
PAQR9, PCOLCE2		rs11709868	3	142,930,002		intergenic						4E−14	[20]
PCOLCE2, PAQR9		rs9832727	3	142,930,268		intergenic						2E−11; 3E−20	[21][22]
ZIC4, RPL21P71		rs113270900	3	147,316,110		intergenic						1E−8	[22]
RPL32P8, TM4SF1-AS1		rs7645585	3	149,406,996		intergenic						1E−8	[22]
SLC33A1		rs76440173 (rs138283229)	3	155,828,335		missense	c.1525G>A	p.Gly509Ser	conflicting	probably damaging	deleterious	5E−9	[22]
IFT80		rs4616688	3	160,324,671		intronic						5E−13	[22]
TNIK, SLC2A2		rs6785233	3	171,039,196		intergenic						3E−8; 4E−15	[20][22]
HGFAC	*LRPAP1*	rs13108218	4	3,442,204		intronic						2E−26; 2E−16; 1E−43	[20][21][22]
DOK7		rs6831256	4	3,471,412		intronic						2E−8	[12]
SORCS2		rs4689653	4	7,221,592		intronic						6E−14	[22]
SORCS2		rs576573069	4	7,223,886		intronic						3E−9	[21]

Mapped gene[a]	Region	SNPid[b]	Chr	Position[c]	LD[d]	Type[e]	cDNA	Protein	ClinVar[f]	Polyphen[g]	Sift[h]	p-value	References
SH3TC1		rs2002574	4	8,222,549		intronic						2E−9	[22]
RBM47, RNU7-74P		rs203273	4	40,416,653		intergenic						5E−12	[22]
TMPRSS11E, UGT2B29P		rs9884390	4	68,507,689		intergenic						7E−24	[21]
UGT2B17, UGT2B15		rs4860987	4	68,625,566		intergenic						6E−49	[22]
UGT2B28 - UGT2B4		chr4:69455038	4	69,455,038		intergenic						4E−27	[20]
ADAMTS3		rs151193598	4	72,437,677		intronic						3E−10	[15]
ADAMTS3 - COX18		rs190671241	4	72,708,425		intergenic						2E−11	[15]
ADAMTS3 - COX18		chr4:72754788	4	72,754,788		intergenic						2E−6	[15]
ADAMTS3 - COX18		rs181948526	4	72,902,905		intergenic						9E−13	[15]
ADAMTS3 - COX18		chr4:72954415	4	72,954,415		intergenic						2E−6	[15]
ANKRD17		chr4:73134560	4	73,134,560		intronic						1E−7	[15]
ANKRD17		rs187918276	4	73,167,847		intronic						3E−13	[15]
ALB, ANKRD17		rs72663045	4	73,311,680		intergenic						6E−7	[21]
LINC02499		rs113759232	4	73,522,447		intergenic						2E−11	[15]
UMLILO, RASSF6		rs573930512	4	73,646,563		intergenic						8E−12	[15]
MTHFD2L		chr4:74133592	4	74,133,592		intronic						3E−6	[15]
MTHFD2L		rs182616603	4	74,219,015		intronic						2E−12	[13]
MTHFD2L		chr4:74265673	4	74,265,673		intronic						5E−12	[15]
AREG - BTC		chr4:74653273	4	74,653,273		intergenic						3E−8	[15]
BTC		chr4:74793806	4	74,793,806		intronic						2E−8	[15]
PARM1		chr4:74990001	4	74,990,001		intronic						8E−9	[15]
PARM1 - RCHY1		chr4:75242737	4	75,242,737		intergenic						6E−9	[15]
PARM1 - RCHY1		chr4:75422391	4	75,422,391		intergenic						1E−7	[15]
CDKL2		chr4:75583178	4	75,583,178		intronic						3E−6	[15]
G3BP2		chr4:75684215	4	75,684,215		intronic						4E−6	[15]
FGF5, PRDM8		rs1458038	4	80,243,569		intergenic						7E−16	[21]
AFF1		rs3775228	4	87,064,014		intronic						2E−12	[20]
ADH1B		rs1229984	4	99,318,162		missense	c.143A>G	p.His48Arg	protective	benign	tolerated	1E−20; 7E−17	[20][21]

Mapped gene[a]	Region	SNPid[b]	Chr	Position[c]	LD[d]	Type[e]	cDNA	Protein	ClinVar[f]	Polyphen[g]	Sift[h]	p-value	References
SLC39A8		rs13107325	4	102,267,552		missense	c.970G>A	p.Ala391Thr	np	benign	tolerated	1E−10; 6E−10	[20][21]
TRIM2		rs41280463	4	153,270,074		intronic						6E−10	[20]
FGB, PLRG1		rs114756490	4	154,555,475		intergenic						1E−11	[20]
FGB		rs6054	4	154,568,456		missense	c.794C>T	p.Pro265Leu	conflicting	probably damaging	tolerated	6E−26	[22]
GUCY1A1		rs990619	4	155,586,526		intergenic						2E−8	[21]
-		rs2716769	4	179,549,287		intergenic						2E−7	[17]
CYP4V2		rs13146272	4	186,199,057		missense	c.775C>A	p.Gln259Lys	benign	benign	tolerated	1E−7	[16]
RAI14		rs146433259	5	34,713,687		intronic						5E−8; 1E−9	[21][22]
IL7R, CAPSL		rs6871748	5	35,885,880		intergenic						5E−8	[22]
ITGA1		rs115421711	5	52,787,392		intergenic						2E−29	[22]
ITGA1, PELO		rs116734477	5	52,799,190		intronic						1E−20; 3E−19	[20][21]
ARL15		rs7735249	5	54,014,309		intronic						2E−8	[22]
C5orf67		rs547065676	5	56,561,722		intergenic						2E−31	[22]
C5orf67		rs3936511	5	56,564,954		intergenic						7E−18	[20]
C5orf67		rs9686661	5	56,565,959		intergenic						1E−8	[21]
LINC02056		rs2925677	5	72,657,802		intergenic						4E−14; 1E−13	[20][21]
LINC02056, RPL7P22		rs3010239	5	72,714,474		intergenic						2E−21	[22]
HMGCR, ANKRD31	*HMGCR*	rs2335418	5	75,307,654		intergenic						5E−321	[22]
HMGCR		rs3843482	5	75,343,434		intronic						2E−45	[13]
HMGCR		rs12654264	5	75,352,778		intronic	c.1368+1176A>T		association			1E−20	[2]
HMGCR		rs3846662	5	75,355,259		intronic						2E−11	[5]
HMGCR, CERT1		rs3846663	5	75,359,901		intronic						8E−12;	[4][19]
HMGCR		rs12916	5	75,360,714		3'-UTR	c.*372T>C		np			2E−15; 5E−45; 1E−11; 8E−78; 1E–-222; 2E−187	[8][9][12] [20][21]

Mapped gene[a]	Region	SNPid[b]	Chr	Position[c]	LD[d]	Type[e]	cDNA	Protein	ClinVar[f]	Polyphen[g]	Sift[h]	p-value	References
CSNK1G3		rs7734476	5	123,513,182		intronic						2E−19; 4E−19	[20][21]
CSNK1G3		rs4530754	5	123,519,722		intronic						4E−12	[12]
SLC22A5		rs546280079	5	132,374,220		intronic						7E−9	[20]
SLC22A5, IRF1-AS1		rs1016988	5	132,408,882		intergenic						1E−12; 5E−11	[16][21]
CYSTM1		rs17118739	5	140,218,325		intronic						3E−9	[20]
HAVCR1, TIMD4	*TIMD4*	rs6882076	5	156,963,286		intergenic						2E−22; 3E−31; 2E−69	[8][12][20]
HAVCR1, TIMD4		rs6874202	5	156,964,617		intergenic						1E−50	[21]
HAVCR1, TIMD4		rs1501908	5	156,971,158		intergenic						1E−11	[4]
HAVCR1		rs1553318	5	157,052,312		intronic						2E−15	[13]
FGFR4		rs351855	5	177,093,242		missense	c.1162G>A	p.Gly388Arg	uncertain	possibly damaging	tolerated	1E−9	[16]
RN7SL554P		rs942922	6	6,972,360		intergenic						2E−8	[22]
ADTRP, AMD1P4		rs147539187	6	11,838,809		intergenic						1E−7	[21]
ADTRP, AMD1P4		rs771191353	6	11,856,024		intergenic						2E−8	[20]
AMD1P4		rs9470298	6	11,868,281		intergenic						5E−18	[22]
MYLIP, MDH1P2	*MYLIP*	rs7746081	6	16,126,703		intergenic						1E−39; 5E−25	[20][21]
MYLIP, MDH1P2		rs3757354	6	16,127,176		intergenic						1E−11; 2E−17; 2E−9	[8][12][13]
MRPL42P2, RNU6-1114P		rs2142672	6	16,196,963		intergenic						2E−8	[9]
HFE	*HFE*	rs1800562	6	26,092,913		missense	c.845G>A	p.Cys282Tyr	conflicting	possibly damaging	deleterious	6E−10; 8E−14; 5E−44	[8][12][20]
HFE		rs79220007	6	26,098,246		3'-UTR	c.*4020T>C		np			2E−48	[21]
MIR3143, RPL10P2		rs71559014	6	27,154,665		intergenic						1E−18	[20]
HIST1H1B		rs201148465	6	27,867,515		synonymous	c.15T>G	p.Ala5=	np		tolerated	3E−8	[16]
ZSCAN16-AS1		rs76079263	6	28,051,887		intergenic						4E−21	[21]
ZSCAN31		rs35814746	6	28,331,311		intronic						4E−16	[20]

Mapped gene[a]	Region	SNPid[b]	Chr	Position[c]	LD[d]	Type[e]	cDNA	Protein	ClinVar[f]	Polyphen[g]	Sift[h]	p-value	References
HLA-H, HCP5B	*HLA*	rs28849176	6	29,880,280		intergenic						1E−17	[21]
TRIM31-AS1, TRIM31		rs9378220	6	30,109,358		intronic						4E−18	[20]
HCG17		rs9391803	6	30,247,600		intronic						2E−19	[21]
HLA-B		rs3179865	6	31,356,417		synonymous	c.369C>T	p.Tyr123=	np		tolerated	2E−19	[21]
MICA, HLA-S		rs7741091	6	31,384,854		intergenic						3E−26	[20]
ATP6V1G2		rs34568880	6	31,546,671		5'-UTR	c.-81G>A		np			6E−10	[21]
FKBPL - PRRT1		chr6:32153409	6	32,153,409		intergenic						3E−6	[15]
HLA-DRA		rs3177928	6	32,444,658		3'-UTR	c.*18G>A		np			2E−15; 3E−17	[8][12]
HLA-DRB9, HLA-DRB5		rs114067101	6	32,490,183		intergenic						8E−13	[13]
HLA-DQA1		rs17205170	6	32,634,706		intronic						1E−44	[20]
MTCO3P1, HLA-DQB1		rs34392107	6	32,696,177		intergenic						6E−45	[21]
COL11A2	*RXRB*	rs2254287	6	33,176,171		intronic			benign			5E−8	[1]
ILRUN		rs76967117	6	34,635,914		intronic						2E−17	[21]
SNRPC		rs114863007	6	34,761,381		intronic						6E−15	[20]
COX6A1P2, RPL12P2		rs913499	6	37,070,656		intergenic						5E−11; 1E−8	[20][21]
KCNK17, KCNK5		rs4711589	6	39,262,795		intergenic						8E−9	[20]
KCNK17, KCNK5		rs55804343	6	39,267,131		intergenic						7E−8	[21]
CNPY3, RPL24P4		rs9471975	6	42,951,484		intergenic						1E−9	[21]
LINC02537, VEGFA		rs998584	6	43,790,159		intergenic						2E−10	[20]
TRAM2-AS1		rs2239619	6	52,588,422		intergenic						4E−15	[16]
LINC01564, KLHL31		rs12665537	6	53,644,654		intergenic						3E−11	[20]
PRDX2P4		rs9496567	6	100,154,877		intergenic						6E−13	[21]
PRDX2P4		rs17185536	6	100,173,055		intergenic						4E−15	[20]
PRDM1, RN7SKP211		rs4946713	6	105,926,140		intergenic						2E−8	[20]
FRK		rs9488822	6	115,991,730		intronic						3E−9; 2E−7	[8][12]
FRK		rs3822855	6	115,995,719		intronic						4E−17	[21]
FRK		rs35936182	6	116,023,963		intronic						1E−18	[20]

Mapped gene[a]	Region	SNPid[b]	Chr	Position[c]	LD[d]	Type[e]	cDNA	Protein	ClinVar[f]	Polyphen[g]	Sift[h]	p-value	References
RSPO3		rs141783576	6	127,118,752		5'-UTR	c.-441G>C		np			2E−11	[20]
RSPO3		rs9491699	6	127,150,388		intronic						2E−11	[21]
L3MBTL3		rs12197047	6	130,068,066		intronic						2E−9; 1E−9	[20][21]
HBS1L		rs7776054	6	135,097,778		intronic						7E−12	[21]
HBS1L		rs9389268	6	135,098,493		intronic						3E−15	[20]
REPS1, ABRACL		rs66883945	6	138,996,691		intergenic						3E−10	[20]
FNDC1 - SOD2	*SLC22A1 - LPA*	rs12191504	6	159,537,803		intergenic						3E−8	[20]
IGF2R		rs73025516	6	160,099,774		intronic						9E−10	[21]
SLC22A1		rs12208357	6	160,122,116		missense	c.181C>T	p.Arg61Cys	np	probably damaging	deleterious	3E−44	[21]
SLC22A1		rs2297374	6	160,154,953		intronic						5E−6	[13]
SLC22A1		rs146534110	6	160,157,037		intronic						9E−14	[21]
SLC22A1		rs1564348	6	160,157,828		intronic						2E−17; 3E−21; 2E−9	[8][12][19]
LPAL2		rs117733303	6	160,501,838		intronic						3E−27	[21]
LPA		rs118039278	6	160,564,494		intronic						6E−170; 2E−102	[20][21]
LPA		rs10455872	6	160,589,086		intronic	c.3947+467T>C		drug response			3E−321	[22]
LPA, PLG		rs186696265	6	160,690,668		intergenic						4E−14	[13]
C7orf50		rs10272002	7	1,007,979		intronic						3E−19	[22]
C7orf50		rs869412	7	1,034,498		intronic						1E−8	[21]
C7orf50		rs10275712	7	1,043,282		intronic						6E−12	[20]
RAC1		rs836550	7	6,400,806		intronic						5E−8	[21]
AHR		rs6968865	7	17,247,645		5'-UTR			np			4E−10	[22]
SP4, RNU1-15P	*DNAH11*	rs28406917	7	21,409,833		intergenic						2E−8	[21]
DNAH11		rs56130071	7	21,559,135		intronic						2E−14; 5E−39	[13][21]
DNAH11		rs150169808	7	21,562,042		intronic						7E−50; 2E−87	[20][22]

Mapped gene[a]	Region	SNPid[b]	Chr	Position[c]	LD[d]	Type[e]	cDNA	Protein	ClinVar[f]	Polyphen[g]	Sift[h]	p-value	References
DNAH11	*DNAH11*	rs12670798	7	21,567,734		intronic						6E−9; 7E−10; 5E−14	[5][8][12]
MIR148A		rs4722551	7	25,952,206		intergenic						4E−14; 2E−19; 1E−17; 6E−37	[12][20][21][22]
JAZF1		rs12055996	7	28,152,174		intronic						4E−9	[22]
MARK2P13, EEPD1		rs67050321	7	36,129,593		intergenic						6E−13; 2E−11; 5E−20	[20][21][22]
INHBA-AS1		rs142220572	7	41,710,893		intergenic						2E−8	[22]
NPC1L1	*NPC1L1*	rs2072183	7	44,539,581		synonymous	c.816C>G	p.Leu272=	benign		tolerated	4E−11; 7E−16	[8][12]
NPC1L1		rs41279633	7	44,541,277		5'-UTR	c.-18C>A		np			1E−10	[13]
NPC1L1, DDX56		rs2073547	7	44,542,732		intergenic						5E−50; 2E−40; 4E−88	[20][21][22]
MLXIPL		rs799157	7	73,605,971		synonymous	c.759A>G	p.Ser253=	np		tolerated	9E−13	[20]
POR		rs2302429	7	75,985,459		intronic						1E−11	[20]
ABCB4		rs31674	7	87,439,148		intronic						6E−9	[20]
ABCB4		rs4148826	7	87,445,103		intronic						7E−9	[21]
BAIAP2L1		rs112758337	7	98,347,956		intronic						2E−9	[21]
PPIAP82, BAIAP2L1		rs377584195	7	98,405,352		intergenic						1E−12	[20]
MOSPD3, TFR2		rs10953298	7	100,619,150		intergenic						2E−17	[20]
EPO, ZAN		rs111338114	7	100,732,869		intergenic						5E−9	[21]
RPS29P15, MUC3A		rs10231941	7	100,934,914		intergenic						3E−13	[21]
PRKAR2B		rs257377	7	107,160,643		3'-UTR	c.*1061G>T		np			2E−9	[22]
PPP1R3A		rs12705932	7	113,987,029		intronic						1E−6	[17]
CAPZA2		rs41785	7	116,845,966		intronic						9E−9	[22]
ZNF800		rs62621812	7	127,375,029		missense	c.307C>T	p.Pro103Ser	np	benign	tolerated	3E−8	[22]
CEP41		chr7:130438531	7	130,438,531		intronic						4E−11	[20]
KLF14, H4P1		rs35363532	7	130,760,670		intergenic						3E−22	[22]

Mapped gene[a]	Region	SNPid[b]	Chr	Position[c]	LD[d]	Type[e]	cDNA	Protein	ClinVar[f]	Polyphen[g]	Sift[h]	p-value	References
BPGM, TUBB3P2		rs2347699	7	134,706,212		intergenic						3E−8	[22]
RN7SKP280, INSIG1-DT		rs4374942	7	155,235,097		intergenic						1E−12	[22]
MCPH1-AS1		rs2911987	8	6,707,055		intergenic						3E−11	[22]
RNU6-526P, RNU6-1151P	*PPP1R3B*	rs7012637	8	9,315,699		intergenic						9E−45	[20]
RNU6-1151P, RNU6-526P		rs2169387	8	9,323,885		intergenic						3E−16	[13]
RNU6-526P, RNU6-1151P		rs9987289	8	9,325,848		intergenic						7E−15;<br>9E−24;<br>5E−36	[8][12][21]
RNU6-526P, RNU6-1151P		rs2126259	8	9,327,636		intergenic						7E−12	[9]
RNU6-1151P, RNU6-526P		rs1461729	8	9,329,732		intergenic						3E−6	[19]
TNKS		rs1350559	8	9,510,233		intergenic						2E−10	[21]
NAT2		rs1495741	8	18,415,371		intergenic						3E−28;<br>5E−12	[20][21]
			8	19,966,981		3'-UTR	c.*1671T>C		benign			1E−21	[20]
DMTN		rs7386762	8	22,069,949		intronic						8E−13	[20]
DMTN		rs59328596	8	22,070,716		intronic						9E−10	[21]
KIF13B		rs117139027	8	29,167,426		missense	c.1105C>T	p.Arg369Trp	np	probably damaging	deleterious	4E−15;<br>7E−13	[20][21]
ANK1		rs72638977	8	41,683,104		intronic						1E−8	[21]
TRMT112P7, RP1		rs10102164	8	54,509,054		intergenic						4E−11	[12]
RP1		rs9298506	8	54,524,964		intronic						3E−23;<br>3E−13	[20][21]
			8	58,476,006		intergenic						4E−9;<br>1E−7	[8][12]
			8	58,479,765		intergenic						2E−11	[13]
			8	58,480,714		intergenic						2E−56;<br>3E−46	[20][21]
RAB2A		rs5891768	8	60,563,015		intronic						2E−11	[20]
TMEM70		rs2306486	8	73,976,259		5'-UTR	c.-23C>T		benign			4E−11	[20]
TMEM74 - TRHR		rs4620259	8	108,979,439		intergenic						5E−8	[21]
TRPS1		rs2737245	8	115,646,356		intronic						5E−30	[20]
TRPS1		rs2737265	8	115,655,407		intronic						2E−18	[21]

Mapped gene[a]	Region	SNPid[b]	Chr	Position[c]	LD[d]	Type[e]	cDNA	Protein	ClinVar[f]	Polyphen[g]	Sift[h]	p-value	References
TRIB1 - LRATD2	*TRIB1*	rs2954021	8	125,469,835		intergenic						1E−7	[9]
TRIB1 - LRATD2		rs17321515	8	125,474,167		intergenic						5E−12	[19]
TRIB1 - LRATD2		rs2954029	8	125,478,730		intergenic						3E−29; 2E−50	[8][12]
TRIB1 - LRATD2		rs28601761	8	125,487,789		intergenic						6E−264; 1E−184	[20][21]
TRIB1 - LRATD2		rs6987702	8	125,492,484		intergenic						3E−6	[5]
TRIB1 - LRATD2		rs112875651	8	125,494,452		intergenic						4E−26	[13]
TRIB1 - LRATD2		chr8:126504383	8	126,504,383		intergenic						5E−324	[22]
PTK2		rs11997161	8	140,728,488		intronic						3E−8	[22]
PTK2		rs28768427	8	140,978,586		intronic						1E−8	[21]
PLEC		rs11781667	8	143,966,688		intergenic						4E−18	[20]
PLEC		rs11136341	8	143,969,375		intronic						4E−13; 7E−12	[8][12]
PLEC		rs11786083	8	143,976,190		intronic						7E−13	[21]
PARP10		rs1134027	8	143,977,246		3'-UTR	c.*238C>T		np			2E−34	[22]
PARP10		rs11784833	8	143,989,244		intronic						2E−10	[13]
VLDLR	*VLDLR*	rs3780181	9	2,640,759		intronic						2E−9; 2E−14; 3E−11; 1E−23	[12][20][21] [22]
BNC2		rs10810657	9	16,884,588		intergenic						1E−11; 5E−20	[20][22]
DENND4C		rs34150222	9	19,313,915		intronic						2E−12	[20]
RPS6		rs67710536	9	19,376,257		3'-UTR	c.*36T>G		np			1E−10	[16]
CDKN2B-AS1		rs6475606	9	22,081,851		intergenic						2E−22	[21]
PCSK5		rs1571790	9	76,114,260		intronic						2E−11	[20]
PCSK5		rs6560499	9	76,115,850		intronic						8E−9	[21]
ABCA1	*ABCA1*	rs2066714	9	104,824,472		missense	c.2649A>G	p.Ile883Met	benign	benign	tolerated	2E−11	[21]
ABCA1		rs11789603	9	104,884,738		intronic						1E−13	[21]
ABCA1		rs2740488	9	104,899,461		intronic						5E−37; 8E−27	[20][21]

Mapped gene[a]	Region	SNPid[b]	Chr	Position[c]	LD[d]	Type[e]	cDNA	Protein	ClinVar[f]	Polyphen[g]	Sift[h]	p-value	References
PKN3		rs13283282	9	128,703,202		intronic						3E−14; 3E−12	[20][21]
			9	133,266,456		intronic			association			2E−228	[22]
			9	133,271,182		intronic						6E−20	[13]
			9	133,274,295		intronic						9E−96	[21]
			9	133,279,427		intergenic						8E−22	[8]
			9	133,279,427		intergenic						2E−41	[12]
TMEM250 - LHX3		chr9:136138765	9	136,138,765		intergenic						7E−136	[20]
INPP5E, PMPCA		rs10448340	9	136,425,617		intergenic						1E−11; 6E−16	[21][22]
PMPCA, INPP5E		rs34297856	9	136,427,993		intergenic						2E−9	[20]
SEC16A		rs3812594	9	136,474,501		missense	c.3115C>T	p.Arg1039Cys	np	benign	tolerated	1E−12	[16]
RBM17, RPL32P23		rs75641219	10	6,087,347		intergenic						3E−8	[22]
GATA3		rs3824667	10	8,058,162		intronic						3E−8	[22]
TRDMT1, VIM-AS1		rs10795464	10	17,213,096		intergenic						3E−13	[22]
CACNB2		rs1757216	10	18,217,497		intronic						1E−8	[22]
CACNB2		rs11014204	10	18,431,916		intronic						3E−9	[21]
ARHGAP12, RPL34P19		rs201700897 (rs796526078)	10	31,957,010		intronic						1E−10	[22]
MARCHF8		rs7908745	10	45,458,319		missense	c.1642T>C	p.Tyr548His	np	benign	tolerated	6E−11	[22]
SGMS1		rs79828839	10	50,592,671		intronic						2E−8	[21]
A1CF		rs41274050	10	50,814,012		missense	c.1168G>A	p.Gly390Ser	likely benign	probably damaging	tolerated	4E−13	[20]
JMJD1C		rs10761756	10	63,412,568		intronic						1E−9	[20]
REEP3		rs7090758	10	63,575,555		intronic						2E−8	[21]
HK1		rs16926246	10	69,333,636		intronic						2E−18	[20]
HK1		rs17476364	10	69,334,748		intronic						9E−11	[21]
CYP26A1, NIP7P1		rs2068888	10	93,079,885		intergenic						6E−32; 5E−20; 2E−46	[20][21][22]
PKD2L1		rs603424	10	100,315,722		intronic						3E−11	[20]

Mapped gene[a]	Region	SNPid[b]	Chr	Position[c]	LD[d]	Type[e]	cDNA	Protein	ClinVar[f]	Polyphen[g]	Sift[h]	p-value	References
GPAM		rs1129555	10	112,150,963		3'-UTR	c.*2587T>C		np			5E−7	[13]
GPAM		rs2250802	10	112,161,596		intronic						6E−15	[21]
GPAM		rs2792735	10	112,162,067		intronic						6E−19	[20]
GPAM		rs2255141	10	112,174,128		intronic						2E−9; 1E−13	[8][12]
ADRB1		rs72823013	10	114,026,474		intergenic						2E−10	[22]
ADRB1		rs72823020	10	114,038,188		intergenic						6E−12	[21]
PNLIPRP2		rs4751996	10	116,638,383		missense	c.1084G>A	p.Val361Ile	np	benign	tolerated	4E−10;1E−17	[20][22]
PNLIPRP2		rs10885997	10	116,638,460		synonymous	c.1161A>G	p.Ser387=	np		tolerated	7E−9	[16]
FAM24B		rs1891110	10	122,850,511		missense	c.5C>T	p.Pro2Leu	np	benign	tolerated	2E−31	[16]
C10orf88, FAM24A		rs10794579	10	122,927,140		intergenic						7E−20; 2E−25	[20][22]
C10orf88		rs12246352	10	122,945,791		intronic						7E−14	[21]
CEND1, GATD1		rs61876729	11	778,857		intergenic						6E−10	[22]
NAP1L4		rs61871243	11	2,977,392		intronic						7E−11	[22]
TRIM6-TRIM34, TRIM5		rs7108486	11	5,655,928		intronic						2E−8	[21]
TRIM5		rs11601507	11	5,679,844		missense	c.334G>A	p.Val112Phe	np	benign	deleterious	3E−24; 2E−15; 3E−40	[20][21][22]
COPB1		rs769117329	11	14,477,390		intronic						2E−8	[22]
SPTY2D1		rs10128711	11	18,611,437		intronic						5E−13	[20]
SPTY2D1		rs10500834	11	18,617,165		intronic						1E−28	[22]
SPTY2D1, SRSF3P1		rs10832963	11	18,642,694		intergenic						4E−13	[21]
CSRP3-AS1, NAV2		rs4756996	11	19,333,933		intergenic						5E−7	[17]
LUZP2 - ANO3		rs1489502	11	26,061,460		intergenic						3E−9	[22]
THEM7P		rs79953563	11	32,131,922		intronic						9E−13	[22]
FADS1	*FADS1*	rs174546	11	61,802,358		3'-UTR	c.*53G>A		np			1E−7; 1E−21; 2E−39	[6][8][12]
FADS1		rs174547	11	61,803,311		intronic						6E−9	[19]
FADS1		rs174551	11	61,806,212		intronic						2E−27	[13]

Mapped gene[a]	Region	SNPid[b]	Chr	Position[c]	LD[d]	Type[e]	cDNA	Protein	ClinVar[f]	Polyphen[g]	Sift[h]	p-value	References
FADS2	*FADS1*	rs174564	11	61,820,833		intronic						6E−63; 3E−48	[20][21]
FADS2		rs174570	11	61,829,740		intronic						4E−13	[5]
NAA40		rs11373615	11	63,941,561		intronic						4E−8	[20]
RELA		rs11227247	11	65,655,382		intronic						6E−9	[21]
MRPL11		rs550317996	11	66,461,002		intronic						2E−10	[20]
ZDHHC24, BBS1		rs74869459	11	66,529,098		intronic						3E−12	[21]
PDGFD		rs115739682	11	103,999,912		intronic						1E−9	[21]
PDGFD		rs10791660	11	104,000,311		intronic						4E−11	[20]
TMPRSS5, MTRF1LP1		rs45543538	11	113,707,269		intergenic						2E−8	[20]
LINC02702, BUD13	*APOA1-C3-A4-A5*	rs12272004	11	116,733,008		intergenic						5E−13	[5]
LINC02702, BUD13		rs1558861	11	116,736,721		intergenic						2E−6	[9]
ZPR1 (ZNF259)		rs964184	11	116,778,201		3'-UTR	c.*724C>G		np			1E−26; 2E−26; 4E−18; 2E−163; 2E−78; 2E−316	[8][12][13] [20][21][22]
IFT46		rs7110984	11	118,571,519		intronic						9E−9	[20]
UBASH3B, GLULP3		rs6589939	11	122,647,817		intergenic						7E−10	[21]
UBASH3B, GLULP3		rs7941030	11	122,651,667		intergenic						3E−13	[20]
ST3GAL4	*ST3GAL4*	rs112771035	11	126,355,981		intronic						8E−55	[20]
ST3GAL4		rs1893351	11	126,357,131		intronic						4E−10	[22]
ST3GAL4		rs59379014	11	126,358,105		intronic						3E−43	[21]
ST3GAL4		rs11220462	11	126,374,057		intronic						1E−15; 7E−21	[8][12]
ST3GAL4		rs4307732	11	126,375,060		intronic						3E−12	[13]
B4GALNT3		rs35882350	12	513,963		intronic						2E−14; 5E−9; 2E−18	[20][21][22]
CCND2		rs76895963	12	4,275,678		intronic						9E−11; 1E−21	[20][22]

Mapped gene[a]	Region	SNPid[b]	Chr	Position[c]	LD[d]	Type[e]	cDNA	Protein	ClinVar[f]	Polyphen[g]	Sift[h]	p-value	References
CD163L1, CD163		rs117692263	12	7,472,418		intronic						2E−8	[22]
LINC00612, VDAC2P2		rs881376	12	9,050,157		intergenic						5E−12	[20]
LINC00612, VDAC2P2		rs201319146	12	9,053,207		intergenic						2E−20	[22]
ETV6		rs117864888	12	11,744,963		intronic						3E−8	[22]
KRAS		rs12318598	12	25,254,792		intergenic						3E−11	[22]
ITPR2		rs1007938	12	26,649,616		intronic						1E−8	[21]
ITPR2		rs111626763	12	26,681,872		splice region			np			2E−8	[22]
ERGIC2		rs10843391	12	29,355,709		intronic						3E−8	[22]
RESF1		rs4931005	12	31,992,066		intronic						2E−8	[22]
SLC2A13		rs10877955	12	40,027,315		intronic						5E−13	[22]
SLC2A13, LINC02555		rs2253736	12	40,139,535		intergenic						2E−9	[20]
LIMA1		rs2160994	12	50,256,274		intronic						7E−15; 4E−17	[20][21]
SCN8A		chr12:51779544	12	51,779,544		intronic						2E−11	[20]
R3HDM2		rs10649122	12	57,256,817		intronic						2E−12	[20]
RAB21		rs61754230	12	71,785,666		missense	c.671C>T	p.Ser224Phe	np	probably damaging	deleterious	4E−12; 4E−15; 1E−8	[16][20][21]
SLC17A8		rs749921369	12	100,395,327		intergenic						7E−11	[20]
WASHC4 (KIAA1033)		rs1663564	12	105,152,394		missense	c.2701G>A	p.Val902Ile	np	benign	tolerated	1E−8	[20]
MVK		rs71079573	12	109,583,090		intronic						3E−8	[20]
ATXN2		rs597808	12	111,535,554		intronic						4E−23; 2E−38	[20][21]
BRAP, ATXN2-AS		rs11065987	12	111,634,620		intergenic						2E−9; 1E−11	[8][12]
RPH3A		rs233721	12	112,593,739		intronic						2E−18	[21]
RPH3A		rs233716	12	112,602,139		intronic						5E−13	[20]
KSR2		rs4767631	12	117,873,938		intronic						6E−7	[7]
			12	120,951,159		intergenic						2E−8	[4]

Mapped gene[a]	Region	SNPid[b]	Chr	Position[c]	LD[d]	Type[e]	cDNA	Protein	ClinVar[f]	Polyphen[g]	Sift[h]	p-value	References
HNF1A	*HNF1A*	rs1169288	12	120,978,847		missense	c.79A>C	p.Ile27Leu	benign	benign	tolerated	1E−15; 6E−21; 4E−45; 3E−76	[8][12][20][22]
HNF1A		rs1169294	12	120,988,791		intronic						2E−27	[21]
C12orf43		rs1169314	12	121,005,313		intronic						2E−8	[13]
DNAH10		rs11057353	12	123,781,140		missense	c.499T>C	p.Ser167Pro	benign	benign	tolerated	4E−12	[20]
SCARB1		rs112403212	12	124,818,708		intronic						1E−8	[21]
SCARB1		rs11057830	12	124,822,507		intronic						2E−15	[20]
FBRSL1		rs12826964	12	132,564,267		intronic						5E−9	[20]
HMGB1, UBE2L5		rs1331698	13	30,442,842		intergenic						8E−11	[22]
BRCA2		rs4942486	13	32,379,251		intronic	c.8755-66T>C		benign			2E−11	[12]
BRCA2, IFIT1P1		rs2238162	13	32,385,062		intronic						4E−15	[21]
PDS5B - KL		chr13:32976656	13	32,976,656		intergenic						8E−27; 4E−47	[20][22]
SETDB2		rs11386165	13	49,474,266		intronic						5E−8	[22]
DLEU1		rs201796	13	50,381,960		intergenic						2E−8	[20]
DLEU1		rs67854369	13	50,471,162		intergenic						4E−9	[22]
KLF12		rs112679104	13	74,132,814		intronic						6E−10	[22]
COL4A2		rs551473284	13	110,385,978		intronic						2E−9; 1E−9	[20][21]
COL4A2		rs75816352	13	110,385,979		intronic						7E−14	[22]
GAS6-AS1, GAS6		rs7140110	13	113,841,051		intronic						2E−28	[20]
GAS6-AS1, GAS6		rs6602912	13	113,843,576		intronic						6E−22	[21]
GAS6		rs6602909	13	113,849,020		intronic						3E−47	[22]
			14	24,402,720		intronic						2E−8; 9E−23; 1E−19; 5E−43	[13][20][21][22]
			14	24,414,681		missense	c.2932G>A	p.Ala978Thr	np	benign	tolerated	4E−11; 3E−15	[8][12]
HECTD1 - HEATR5A		rs139262716	14	31,255,905		intergenic						1E−8	[22]
CFL2, RPL12P6		rs11846704	14	34,717,488		intergenic						3E−9	[22]

Mapped gene[a]	Region	SNPid[b]	Chr	Position[c]	LD[d]	Type[e]	cDNA	Protein	ClinVar[f]	Polyphen[g]	Sift[h]	p-value	References
RPL12P6, CFL2		rs10151517	14	34,718,046		intergenic						5E−8	[20]
SYNE2		rs8008068	14	63,766,999		intronic						4E−8	[21]
ESR2		chr14:64236436	14	64,236,436		intronic						7E−12; 4E−17	[20][22]
ADAM21P1, COX16		rs8005362	14	70,304,150		intergenic						2E−25	[22]
ADAM20, MED6		rs6573971	14	70,544,752		intergenic						3E−10	[21]
RN7SL77P, TTC9		rs9646133	14	70,629,627		intergenic						5E−22	[16]
MAP3K9 - PCNX1		chr14:70874146	14	70,874,146		intergenic						4E−14	[20]
ZFYVE1		rs61988556	14	72,972,550		intronic						2E−9	[21]
MIDEAS		rs13379043	14	73,783,423		intronic						2E−6; 4E−13; 8E−24	[16][20][22]
			14	94,301,859		intergenic						4E−12	[21]
			14	94,378,610		missense	c.1096G>A	p.Glu366Lys	pathogenic	probably damaging	deleterious	1E−21	[16]
			14	94,380,925		missense	c.863A>T	p.Glu288Val	conflicting	probably damaging	deleterious	2E−15; 4E−30	[20][22]
LINC00637, ATP5MJ		rs12891477	14	103,866,422		intergenic						3E−12	[22]
SECISBP2L		rs7164309	15	49,025,561		intronic						3E−9	[20]
FAM227B		rs10851478	15	49,536,822		intronic						6E−9	[21]
FAM227B		rs566865466	15	49,550,054		intronic						6E−12	[22]
WDR72		rs79391862	15	53,447,229		intergenic						9E−11	[22]
			15	57,220,086		intronic						2E−9	[21]
			15	57,240,500		intronic						4E−17	[20]
			15	58,391,167		intronic						9E−16	[21]
			15	58,430,392		intronic						1E−43	[22]
			15	58,434,545		intronic						9E−18	[21]
ADAM10		chr15:58679807	15	58,679,807		intronic						2E−30	[20]
C2CD4A, NPM1P47		rs72749770	15	62,073,266		intergenic						9E−10	[22]
TPM1		rs76162994	15	63,036,763		intergenic						4E−8	[22]
USP3, LINC02568		rs11636087	15	63,496,068		intergenic						2E−17	[22]

Mapped gene[a]	Region	SNPid[b]	Chr	Position[c]	LD[d]	Type[e]	cDNA	Protein	ClinVar[f]	Polyphen[g]	Sift[h]	p-value	References
USP3, LINC02568		rs62011285	15	63,498,864		intergenic						2E−8	[21]
USP3, LINC02568		rs56369308	15	63,500,058		intergenic						1E−12	[20]
LMAN1L		rs112987086	15	74,814,378		intronic						1E−11	[21]
LMAN1L		rs12917376	15	74,823,826		intronic						4E−8	[20]
SCAMP2, MPI		rs9673065	15	74,888,551		intergenic						2E−11	[22]
CRTC3, IQGAP1		rs56402930	15	90,529,730		intergenic						8E−6	[21]
CRTC3		rs6496691	15	90,566,379		intronic						2E−9	[22]
PCSK6		rs34631529	15	101,305,277		missense	c.2891C>T	p.Thr964Met	likely benign	probably damaging	deleterious	6E−9	[22]
LITAF		rs12445804	16	11,612,244		intronic						7E−14; 8E−9; 5E−23	[20][21][22]
Y_RNA, MIR193BHG		rs246179	16	14,287,318		intergenic						8E−9	[22]
STX1B		rs12920772	16	31,001,460		intronic						8E−11	[20]
FTO		rs62033400	16	53,777,876		intronic						1E−11	[21]
CETP, HERPUD1	*CETP*	rs247617	16	56,956,804		intergenic						2E−24	[13]
HERPUD1, CETP		rs183130	16	56,957,451		intergenic			association			8E−59	[20]
HERPUD1, CETP		rs3764261	16	56,959,412		intergenic			no interp.			9E−13; 2E−34; 1E−49	[8][12][21]
WWP2		rs78432537	16	69,839,928		intronic						5E−9	[20]
HYDIN		rs56212732	16	70,896,467		intronic						2E−9	[21]
HYDIN		rs145090930	16	70,922,412		intronic						1E−11	[20]
MARVELD3, TAT-AS1	*TNXL4B*	rs9929977	16	71,605,150		intergenic						1E−14	[21]
DHODH		rs11648003	16	72,018,449		intronic						2E−20	[13]
TXNL4B, HPR		rs34042070	16	72,067,626		intronic						5E−105; 2E−73; 1E−177	[20][21][22]
HPR, TXNL4B		rs2000999	16	72,074,194		intronic						2E−22; 4E−41	[8][12]
PMFBP1, LINC01572		rs7202323	16	72,183,214		intergenic						8E−25	[21]
MAF		rs1862719	16	79,470,160		intronic						8E−11	[22]

Mapped gene[a]	Region	SNPid[b]	Chr	Position[c]	LD[d]	Type[e]	cDNA	Protein	ClinVar[f]	Polyphen[g]	Sift[h]	p-value	References
OSGIN1, MLYCD		rs4782568	16	83,946,924		intronic						8E−15; 6E−15; 1E−24	[20][21][22]
ZFPM1		rs139829677	16	88,502,880		intronic						1E−13	[22]
ZFPM1		rs147032017	16	88,514,388		synonymous	c.270C>T	p.Asp90=	np		tolerated	5E−9	[16]
ZFPM1-AS1, ZFPM1		rs77013160	16	88,516,463		intronic						6E−9; 2E−8	[20][21]
WDR81		rs11431553	17	1,719,561		intronic						3E−10	[22]
DPH1		rs7207466	17	2,039,567		intronic						6E−10	[22]
ASGR1		rs55714927	17	7,176,997		synonymous	c.267G>A	p.Lys89=	np		tolerated	9E−12; 7E−37; 4E−23	[13][20][21]
DLG4		rs314253	17	7,188,331		3'-UTR	c.*2377A>G		np			3E−10	[12]
TP53		rs9894946	17	7,667,762		intronic						2E−9	[21]
RAI1		rs71367412	17	17,717,693		intronic						6E−9	[20]
VTN		rs704	17	28,367,840		missense	c.1199C>T	p.Thr400Met	np	benign	tolerated	5E−29; 3E−15; 9E−13	[16][20][21]
TAOK1, ABHD15		rs56208742	17	29,557,649		intergenic						1E−11; 2E−8	[20][21]
NF1		rs12603885	17	31,139,704		intronic						3E−14; 1E−14	[20][21]
NF1		rs11080150	17	31,302,308		intronic						1E−7	[16]
STAT5A		rs28727898	17	42,297,600		intronic						2E−9	[20]
EFCAB13	*OSBPL7*	rs7206971	17	47,347,749		intronic						4E−9; 3E−7; 4E−10	[8][12][13]
NPEPPS		rs36043200	17	47,552,040		intronic						1E−36	[21]
NPEPPS		rs10445374	17	47,585,017		intronic						2E−46	[20]
LINC02086		rs3110609	17	48,676,181		intergenic						3E−12	[21]
LINC02086		rs3096644	17	48,680,213		intergenic						3E−13	[20]
APOH	*APOH*	rs1801689	17	66,214,462		missense	c.973T>G	p.Cys325Gly	np	probably damaging	deleterious	1E−11; 1E−30; 2E−24	[12][20][21]
ARSG	*ABCA6*	rs12936113	17	68,404,922		intronic						2E−8	[21]

Mapped gene[a]	Region	SNPid[b]	Chr	Position[c]	LD[d]	Type[e]	cDNA	Protein	ClinVar[f]	Polyphen[g]	Sift[h]	p-value	References
ABCA6	*ABCA6*	rs77542162	17	69,085,137		missense	c.4075T>C	p.Cys1359Arg	np	probably damaging	deleterious	2E−18; 2E−98; 6E−74; 3E−169	[13][20][21][22]
ABCA10		rs72631343	17	69,195,129		intronic						5E−21	[21]
H3-3B		rs73352129	17	75,782,528		intronic						1E−13	[20]
UNK		rs2125345	17	75,786,110		intronic						3E−22	[16]
TRIM65		rs56000661	17	75,882,573		intronic						1E−23	[22]
PGS1		rs12948394	17	78,386,710		intronic						5E−15; 5E−18; 2E−25	[20][21][22]
FOXK2		rs3736206	17	82,572,553		intronic						2E−9	[22]
B3GNTL1		rs111571253	17	83,051,228		intronic						1E−8	[22]
CTAGE1		rs79588679	18	22,327,807		intergenic						4E−8	[13]
NPC1	*NPC1*	rs2510344	18	23,533,321		intronic	c.3754+34A>G		benign			2E−9	[22]
TAF4B		rs12960731	18	26,338,309		intronic						8E−7	[17]
LIPG	*LIPG*	rs77960347	18	49,583,585		missense	c.1187A>G	p.Asn396Ser	np	probably damaging	tolerated	6E−14; 8E−15; 3E−24	[20][21][22]
LIPG, SMUG1P1		rs7241918	18	49,634,583		intergenic						1E−8	[21]
ATP8B1		rs369298568	18	57,651,800		intronic						5E−12	[22]
THOP1		rs2741991	19	2,802,094		intronic						3E−9	[20]
THOP1		rs1640273	19	2,803,229		intronic						4E−11	[22]
HDGFL2		rs111174163	19	4,492,942		intronic						9E−10	[22]
S1PR5, ATG4D	*LDLR*	rs549956721	19	10,526,288		intergenic						2E−10	[21]
DNM2		chr19:10734951	19	10,734,951		intronic						7E−10	[15]
YIPF2 - SMARCA4		chr19:10948031	19	10,948,031		intergenic						1E−11	[15]
SMARCA4		rs1122608	19	11,052,925		intronic						1E−14	[19]
SMARCA4		rs10423733	19	11,075,243		intronic						3E−428	[20]
SMARCA4		rs143020224	19	11,076,648		intronic						9E−605	[21]
SMARCA4		rs112374545	19	11,078,223		intronic						7E−142	[13]

Mapped gene[a]	Region	SNPid[b]	Chr	Position[c]	LD[d]	Type[e]	cDNA	Protein	ClinVar[f]	Polyphen[g]	Sift[h]	p-value	References
			19	11,084,354		intronic						2E−7	[6]
			19	11,091,630		intronic	c.67+2015G>T		benign			2E−26; 2E−51; 4E−26; 4E−117; 4E−262	[1][2][4][8] [12]
			19	11,100,236		synonymous	c.81C>T	p.Cys27=	benign		tolerated	4E−14	[5]
			19	11,116,804		intronic	c.1706-55A>C		benign			4E−89	[21]
			19	11,127,797		intronic						7E−6	[9]
			19	11,198,502		intergenic						2E−23	[15]
			19	11,224,801		intronic						6E−16	[21]
RGL3		chr19:11398422	19	11,398,422		intronic						5E−15	[15]
ECSIT		chr19:11507217	19	11,507,217		intronic						3E−8	[15]
ZNF441 - ZNF491		chr19:11789826	19	11,789,826		intergenic						2E−7	[15]
ZNF788P, ZNF20		rs117590032	19	12,095,815		intronic						5E−8	[20]
CYP4F12		rs4808360	19	15,687,205		intronic						8E−10	[21]
PDE4C		rs62120394	19	18,227,899		intronic						6E−12	[21]
PDE4C		rs8112975	19	18,230,100		intronic						6E−12	[20]
			19	19,259,532		intronic						3E−11	[14]
			19	19,268,740		missense	c.499G>A	p.Glu167Lys	np	possibly damaging	tolerated	3E−186	[20]
			19	19,277,691		intronic						2E−158	[21]
			19	19,285,807		intronic						9E−37	[21]
			19	19,296,909		intronic						2E−8; 7E−22; 1E−11; 3E−54; 3E−8	[4][8][9][12] [19]
			19	19,349,732		intronic						3E−23	[13]
			19	19,394,368		intronic						4E−8	[15]
			19	19,494,483		intronic						2E−7	[15]

Mapped gene[a]	Region	SNPid[b]	Chr	Position[c]	LD[d]	Type[e]	cDNA	Protein	ClinVar[f]	Polyphen[g]	Sift[h]	p-value	References
			19	19,547,663		intergenic						3E−9; 3E−8	[1][2]
			19	19,678,719		intronic						3E−6	[5]
			19	19,727,152		intronic						3E−8	[15]
ZNF826P		rs144984216	19	20,369,092		intronic						3E−8	[21]
ZNF486 - ZNF737		chr19:20439917	19	20,439,917		intergenic						7E−14	[20]
CYP2A6		rs56113850	19	40,847,202		intronic						2E−10	[21]
CYP2A6		rs56267346	19	40,847,433		intronic						3E−14	[20]
ZNF574		rs201596848	19	42,080,806		missense	c.2200C>T	p.Arg734Cys	np	possibly damaging	tolerated	1E−11	[16]
POU2F2		chr19:42103100	19	42,103,100		intronic						2E−8	[15]
XRCC1		chr19:43545210	19	43,545,210		intronic						1E−7	[15]
XRCC1		rs2021092	19	43,564,554		intronic						9E−10	[21]
XRCC1		rs3213282	19	43,568,728		intronic						3E−11	[20]
CEACAM20		rs62116889	19	44,518,526		intronic						2E−33	[21]
CEACAM20 - IGSF23		chr19:44603140	19	44,603,140		intergenic						3E−7	[15]
IGSF23		rs62119267	19	44,631,381		intronic						9E−295	[20]
CEACAM16-AS1, BCL3		rs1551891	19	44,728,555		intergenic						4E−490	[21]
			19	44,844,654		intergenic						7E−343	[21]
			19	44,888,997		3'-UTR	c.*618C>T		np			1E−711	[21]
			19	44,892,009		intronic						2E−19; 5E−8	[5][6]
			19	44,908,822		missense	c.526C>T	p.Arg176Cys	drug response	probably damaging	deleterious	3E−19; 1E−17	[14][18]
			19	44,909,976		intergenic						5E−324; 5E−50	[13][15]
			19	44,918,393		intronic						4E−323	[22]

Mapped gene[a]	Region	SNPid[b]	Chr	Position[c]	LD[d]	Type[e]	cDNA	Protein	ClinVar[f]	Polyphen[g]	Sift[h]	p-value	References
			19	44,919,689		downstream	c.*459A>G		no interp.			3E−43; 1E−60; 1E−20; 4E−27; 9E−147; 2E−40; 1E−14; 2E−178; 5E−35	[1][2][3][4] [8][9][11] [12][19]
			19	44,987,027		intronic						9E−9	[21]
			19	45,114,005		intronic						5E−17	[21]
			19	45,531,618		intronic						2E−9	[15]
			19	45,791,965		intronic						2E−45	[20]
			19	45,933,306		intergenic						2E−31	[21]
IGFL1, IGFL1P2		rs145725232	19	46,249,284		intergenic						8E−7	[21]
HNRNPMP2, SLC1A5		rs16980741	19	46,792,101		intergenic						3E−8	[20]
FUT2		rs516316	19	48,702,888		intronic						1E−61; 1E−46	[20][21]
ZNF667-AS1		rs61469827	19	56,500,104		intergenic						3E−8	[20]
ZNF329		rs35081008	19	58,150,868		intergenic						3E−23; 1E−27; 9E−35	[20][21][22]
ZNF329, ZNF274		rs117492019	19	58,170,494		intergenic						1E−8	[13]
RBCK1		rs6139104	20	409,666		intronic						2E−11	[22]
GPCPD1		rs73075609	20	5,600,143		intronic						1E−11; 2E−8; 2E−16	[20][21][22]
LINC01723		rs438568	20	12,978,039		intergenic						1E−9; 4E−9; 7E−16	[20][21][22]
LINC01723		rs364585	20	12,982,070		intergenic						4E−10	[12]
BANF2, RNU6-192P		rs61433703	20	17,823,423		intergenic						3E−8	[21]
RNU6-192P		rs2618568	20	17,863,324		intergenic						7E−15	[13]
RNU6-192P		rs2618567	20	17,863,848		intergenic						8E−41; 3E−73	[20][22]

Mapped gene[a]	Region	SNPid[b]	Chr	Position[c]	LD[d]	Type[e]	cDNA	Protein	ClinVar[f]	Polyphen[g]	Sift[h]	p-value	References
RNU6-192P		rs2618566	20	17,864,040		intergenic						1E−29	[21]
RNU6-192P		rs2328223	20	17,865,277		intergenic						6E−9	[12]
ENTPD6		rs1044573	20	25,226,018		3'-UTR	c.*421A>G		np			4E−13	[22]
PYGB, ENTPD6		rs6050463	20	25,228,354		intergenic						6E−9;<br>2E−9	[20][21]
EIFE2S2 - ASIP		chr20:34120437	20	34,120,437		intergenic						4E−21	[20]
FER1L4		rs224424	20	35,560,231		intergenic						3E−16	[21]
DHX35 - MAFB		chr20:38966760	20	38,966,760		intronic						5E−8	[15]
DHX35 - MAFB		chr20:39116691	20	39,116,691		intergenic						4E−8	[15]
LOC105372618		rs2902940	20	40,462,847		intronic						1E−8;<br>2E−11	[8][12]
DHX35 - MAFB		rs1883711	20	40,551,182		intergenic						4E−16;<br>4E−96;<br>1E−63;<br>4E−171	[13][20][21]<br>[22]
DHX35 - MAFB		rs191064657	20	40,563,663		intergenic						9E−10	[21]
DHX35 - MAFB		rs6102059	20	40,600,144		intergenic						4E−9	[4]
TOP1		rs6029526	20	41,043,978		intronic						3E−19;<br>5E−18	[8][12]
PLCG1		rs6093446	20	41,152,292		intronic						7E−22	[21]
PLCG1		rs753381	20	41,168,825		missense	c.2438T>C	p.Ile813Thr	np	benign	tolerated	7E−8	[19]
CHD6		rs2866745	20	41,606,278		intronic						1E−21	[20]
HNF4A	*HNF4A*	rs1800961	20	44,413,724		missense	c.416C>T	p.Thr139Ile	benign/likely benign	benign	tolerated	5E−28;<br>4E−23	[20][21]
PLTP	*PLTP*	rs6065904	20	45,906,012		intronic						5E−13	[20]
PCIF1, PLTP		rs6073958	20	45,923,216		intergenic						3E−11	[21]
SUMO1P1, BCAS1		rs6022850	20	53,918,861		intergenic						9E−11	[22]
NTSR1		rs3746778	20	62,710,120		intronic						1E−15	[22]
SLC2A4RG		rs2256814	20	63,742,630		intronic						7E−9	[21]
TCEA2		rs6090040	20	64,060,707		intronic						1E−8;<br>8E−19	[20][22]
TCEA2		rs6062343	20	64,064,578		intronic						1E−6	[16]
PCMTD2		rs6090101	20	64,278,167		intronic						3E−11	[21]

Mapped gene[a]	Region	SNPid[b]	Chr	Position[c]	LD[d]	Type[e]	cDNA	Protein	ClinVar[f]	Polyphen[g]	Sift[h]	p-value	References
NRIP1 - USP25		rs62219001	21	15,210,560		intergenic						1E−12	[22]
NRIP1 - USP25		rs12106385	21	15,214,362		intergenic						5E−8	[20]
SCAF4		rs67038483	21	31,723,790		intronic						7E−6	[21]
SCAF4		rs73201545	21	31,733,452		intergenic						8E−13	[22]
DOP1B		rs11911615	21	36,180,834		intronic						5E−10	[22]
HMGN1, BRWD1-AS1		rs4818025	21	39,337,245		intergenic						9E−11	[21]
BRWD1-AS1, HMGN1		rs1963676	21	39,338,034		intergenic						1E−13; 2E−22	[20][22]
PKNOX1		rs2839619	21	43,016,067		intronic						8E−6	[7]
COMT		rs165722	22	19,961,490		intronic						3E−9	[22]
UBE2L3		rs5754102	22	21,561,983		intronic						2E−8	[22]
MTMR3		rs5763662	22	29,982,714		intronic						1E−8	[12]
LIF-AS1, HORMAD2		rs16988410	22	30,221,596		intergenic						7E−9	[22]
HMGXB4		rs35288294	22	35,280,146		intronic						7E−10	[22]
TOM1		rs138730	22	35,300,938		intronic						6E−9	[20]
SLC25A17		rs2076674	22	40,774,059		intronic						3E−12	[16]
XPNPEP3		rs138352	22	40,872,921		intronic						8E−16	[22]
XPNPEP3		rs5758128	22	40,918,008		intronic						6E−13	[20]
Y_RNA, RBX1		rs960596	22	40,997,516		intergenic						1E−9	[21]
PNPLA3		rs2294915	22	43,945,024		intronic						6E−10	[22]
FBLN1		rs13268	22	45,600,418		missense	c.2084A>G	His695Arg	benign	possibly damaging	tolerated	4E−10; 9E−14	[20][22]
			22	46,231,706		intronic						3E−8	[12]
C22orf34, RN7SKP252		rs1807675	22	49,674,346		intergenic						7E−10	[22]
PPP6R2		rs9616822	22	50,402,144		intronic						4E−15	[22]
PPP6R2		rs12162782	22	50,415,197		intronic						9E−10; 4E−9	[20][21]
ARSL (ARSE)		rs35143646	X	2,938,114		missense	c.1270G>A	p.Gly424Ser	benign	possibly damaging	tolerated	2E−25	[22]
EIF1AX		rs73447108	X	20,136,228		intronic						8E−18	[22]
AR		rs5031002	X	67,722,783		intronic						2E−7	[6]

Mapped gene[a]	Region	SNPid[b]	Chr	Position[c]	LD[d]	Type[e]	cDNA	Protein	ClinVar[f]	Polyphen[g]	Sift[h]	p-value	References
TDGF1P3		rs5942956	X	110,520,525		5'-UTR						2E−102	[22]
M6PRP1, CHRDL1		rs144832584	X	110,662,875		intergenic						2E−8	[22]
GDI1, FAM50A		rs188437955	X	154,443,984		intergenic						1E−8	[22]

[a.] Gene affected by the variant, or the closest genes in case of intergenic variants. [b.] Identification in the dbSNPs database (www.ncbi.nlm.nih.gov/snp). [c.] Positions are relative to genome assembly GRCh38. [d.] Linkage disequilibrium (LD) tracks were taken from Berisa and Prickrel (2016) [23]. [e.] Type of mutation. [f.] Classification in the ClinVar database (www.ncbi.nlm.nih.gov/clinvar) of variants affecting coding regions: no interp., no interpretation; np, not present. [g.] Prediction of the functional effect using PolyPhen (genetics.bwh.harvard.edu/pph2/). [h.] Prediction of the functional effect using Sift (sift.bii.a-star.edu.sg/).

Supplementary references for European populations

1. Willer, C.J.; Sanna, S.; Jackson, A.U.; Scuteri, A.; Bonnycastle, L.L.; Clarke, R.; Heath, S.C.; Timpson, N.J.; Najjar, S.S.; Stringham, H.M., et al. Newly identified loci that influence lipid concentrations and risk of coronary artery disease. *Nat Genet* **2008**, *40*, 161-169, doi:10.1038/ng.76.
2. Kathiresan, S.; Melander, O.; Guiducci, C.; Surti, A.; Burtt, N.P.; Rieder, M.J.; Cooper, G.M.; Roos, C.; Voight, B.F.; Havulinna, A.S., et al. Six new loci associated with blood low-density lipoprotein cholesterol, high-density lipoprotein cholesterol or triglycerides in humans. *Nat Genet* **2008**, *40*, 189-197, doi:10.1038/ng.75.
3. Sandhu, M.S.; Waterworth, D.M.; Debenham, S.L.; Wheeler, E.; Papadakis, K.; Zhao, J.H.; Song, K.; Yuan, X.; Johnson, T.; Ashford, S., et al. LDL-cholesterol concentrations: a genome-wide association study. *Lancet* **2008**, *371*, 483-491, doi:10.1016/S0140-6736(08)60208-1.
4. Kathiresan, S.; Willer, C.J.; Peloso, G.M.; Demissie, S.; Musunuru, K.; Schadt, E.E.; Kaplan, L.; Bennett, D.; Li, Y.; Tanaka, T., et al. Common variants at 30 loci contribute to polygenic dyslipidemia. *Nat Genet* **2009**, *41*, 56-65, doi:10.1038/ng.291.
5. Aulchenko, Y.S.; Ripatti, S.; Lindqvist, I.; Boomsma, D.; Heid, I.M.; Pramstaller, P.P.; Penninx, B.W.J.H.; Janssens, A.C.J.W.; Wilson, J.F.; Spector, T., et al. Loci influencing lipid levels and coronary heart disease risk in 16 European population cohorts. *Nature Genetics* **2008**, *41*, 47, doi:10.1038/ng.269.
6. Sabatti, C.; Service, S.K.; Hartikainen, A.L.; Pouta, A.; Ripatti, S.; Brodsky, J.; Jones, C.G.; Zaitlen, N.A.; Varilo, T.; Kaakinen, M., et al. Genome-wide association analysis of metabolic traits in a birth cohort from a founder population. *Nat Genet* **2009**, *41*, 35-46, doi:10.1038/ng.271.
7. Zemunik, T.; Boban, M.; Lauc, G.; Janković, S.; Rotim, K.; Vatavuk, Z.; Bencić, G.; Dogas, Z.; Boraska, V.; Torlak, V., et al. Genome-wide association study of biochemical traits in Korcula Island, Croatia. *Croat Med J* **2009**, *50*, 23-33, doi:10.3325/cmj.2009.50.23.
8. Teslovich, T.M.; Musunuru, K.; Smith, A.V.; Edmondson, A.C.; Stylianou, I.M.; Koseki, M.; Pirruccello, J.P.; Ripatti, S.; Chasman, D.I.; Willer, C.J., et al. Biological, clinical and population relevance of 95 loci for blood lipids. *Nature* **2010**, *466*, 707-713, doi:10.1038/nature09270.
9. Waterworth, D.M.; Ricketts, S.L.; Song, K.; Chen, L.; Zhao, J.H.; Ripatti, S.; Aulchenko, Y.S.; Zhang, W.; Yuan, X.; Lim, N., et al. Genetic variants influencing circulating lipid levels and risk of coronary artery disease. *Arterioscler Thromb Vasc Biol* **2010**, *30*, 2264-2276, doi:10.1161/ATVBAHA.109.201020.
10. Shen, H.; Damcott, C.M.; Rampersaud, E.; Pollin, T.I.; Horenstein, R.B.; McArdle, P.F.; Peyser, P.A.; Bielak, L.F.; Post, W.S.; Chang, Y.P., et al. Familial defective apolipoprotein B-100 and increased low-density lipoprotein cholesterol and coronary artery calcification in the old order amish. *Arch Intern Med* **2010**, *170*, 1850-1855, doi:10.1001/archinternmed.2010.384.
11. Keller, M.; Schleinitz, D.; Förster, J.; Tönjes, A.; Böttcher, Y.; Fischer-Rosinsky, A.; Breitfeld, J.; Weidle, K.; Rayner, N.W.; Burkhardt, R., et al. THOC5: a novel gene involved in HDL-cholesterol metabolism. *J Lipid Res* **2013**, *54*, 3170-3176, doi:10.1194/jlr.M039420.
12. Willer, C.J.; Schmidt, E.M.; Sengupta, S.; Peloso, G.M.; Gustafsson, S.; Kanoni, S.; Ganna, A.; Chen, J.; Buchkovich, M.L.; Mora, S., et al. Discovery and refinement of loci associated with lipid levels. *Nat Genet* **2013**, *45*, 1274-1283, doi:10.1038/ng.2797.
13. Surakka, I.; Horikoshi, M.; Mägi, R.; Sarin, A.P.; Mahajan, A.; Lagou, V.; Marullo, L.; Ferreira, T.; Miraglio, B.; Timonen, S., et al. The impact of low-frequency and rare variants on lipid levels. *Nat Genet* **2015**, *47*, 589-597, doi:10.1038/ng.3300.
14. Southam, L.; Gilly, A.; Süveges, D.; Farmaki, A.E.; Schwartzentruber, J.; Tachmazidou, I.; Matchan, A.; Rayner, N.W.; Tsafantakis, E.; Karaleftheri, M., et al. Whole genome sequencing and imputation in isolated populations identify genetic associations with medically-relevant complex traits. *Nat Commun* **2017**, *8*, 15606, doi:10.1038/ncomms15606.
15. Davis, J.P.; Huyghe, J.R.; Locke, A.E.; Jackson, A.U.; Sim, X.; Stringham, H.M.; Teslovich, T.M.; Welch, R.P.; Fuchsberger, C.; Narisu, N., et al. Common, low-frequency, and rare genetic

variants associated with lipoprotein subclasses and triglyceride measures in Finnish men from the METSIM study. *PLoS Genet* **2017**, *13*, e1007079, doi:10.1371/journal.pgen.1007079.
16. Liu, D.J.; Peloso, G.M.; Yu, H.; Butterworth, A.S.; Wang, X.; Mahajan, A.; Saleheen, D.; Emdin, C.; Alam, D.; Alves, A.C., et al. Exome-wide association study of plasma lipids in >300,000 individuals. *Nat Genet* **2017**, *49*, 1758-1766, doi:10.1038/ng.3977.
17. Lutz, M.W.; Casanova, R.; Saldana, S.; Kuchibhatla, M.; Plassman, B.L.; Hayden, K.M. Analysis of pleiotropic genetic effects on cognitive impairment, systemic inflammation, and plasma lipids in the Health and Retirement Study. *Neurobiol Aging* **2019**, *80*, 173-186, doi:10.1016/j.neurobiolaging.2018.10.028.
18. Tabassum, R.; Rämö, J.T.; Ripatti, P.; Koskela, J.T.; Kurki, M.; Karjalainen, J.; Palta, P.; Hassan, S.; Nunez-Fontarnau, J.; Kiiskinen, T.T.J., et al. Genetic architecture of human plasma lipidome and its link to cardiovascular disease. *Nat Commun* **2019**, *10*, 4329, doi:10.1038/s41467-019-11954-8.
19. Kulminski, A.M.; Loika, Y.; Nazarian, A.; Culminskaya, I. Quantitative and Qualitative Role of Antagonistic Heterogeneity in Genetics of Blood Lipids. *J Gerontol A Biol Sci Med Sci* **2020**, *75*, 1811-1819, doi:10.1093/gerona/glz225.
20. Ripatti, P.; Rämö, J.T.; Mars, N.J.; Fu, Y.; Lin, J.; Söderlund, S.; Benner, C.; Surakka, I.; Kiiskinen, T.; Havulinna, A.S., et al. Polygenic Hyperlipidemias and Coronary Artery Disease Risk. *Circ Genom Precis Med* **2020**, *13*, e002725, doi:10.1161/CIRCGEN.119.002725.
21. Richardson, T.G.; Sanderson, E.; Palmer, T.M.; Ala-Korpela, M.; Ference, B.A.; Davey Smith, G.; Holmes, M.V. Evaluating the relationship between circulating lipoprotein lipids and apolipoproteins with risk of coronary heart disease: A multivariable Mendelian randomisation analysis. *PLoS Med* **2020**, *17*, e1003062, doi:10.1371/journal.pmed.1003062.
22. Klimentidis, Y.C.; Arora, A.; Newell, M.; Zhou, J.; Ordovas, J.M.; Renquist, B.J.; Wood, A.C. Phenotypic and Genetic Characterization of Lower LDL Cholesterol and Increased Type 2 Diabetes Risk in the UK Biobank. *Diabetes* **2020**, *69*, 2194-2205, doi:10.2337/db19-1134.
23. Berisa, T.; Pickrell, J.K. Approximately independent linkage disequilibrium blocks in human populations. **2016**, doi:10.1093/bioinformatics/btv546.

Table S1b: SNVs associated with low-density lipoprotein cholesterol in East-Asian populations.

Mapped gene[a]	Region	SNPid[b]	Chr	Position[c]	Type[d]	cDNA	Protein	ClinVar[e]	Polyphen[f]	Sift[g]	p-value	References
			1	25,422,778	intronic						1E−8	[9]
			1	25,434,429	intronic						2E−18	[5]
PIGV, RN7SL165P		rs12748152	1	26,811,902	intergenic						3E−12	[5]
BSND - PCSK9	*PCSK9*	rs7523141	1	55,033,125	intergenic						3E−25	[7]
BSND - PCSK9		rs7525649	1	55,033,483	intergenic						3E−11	[2]
PCSK9, BSND		rs2479409	1	55,038,977	5'-UTR	c.-861G>A		benign			3E−42	[5]
PCSK9		rs151193009	1	55,043,912	missense	c.277C>T	p.Arg93Cys	conflicting	probably damaging	deleterious	6E−17; 8E−32	[3][9]
PCSK9		rs12136600	1	55,055,522	intronic						4E−20	[7]
PCSK9		rs505151	1	55,063,514	missense	c.2009G>A	p.Gly670Glu		benign	tolerated	8E−7; 9E−9	[5]
USP24 - Y_RNA		chr1:55509585	1	55,509,585	intergenic						4E−36	[10]
LINC01755		rs147943615	1	55,902,364	intergenic						1E−27	[7]
USP1	*DOCK7 - ANGPTL3*	rs10158897	1	62,447,248	intronic						9E−6	[1]
DOCK7 - ANGPTL3		rs636523	1	62,454,337	intergenic						5E−7	[1]
DOCK7		rs11485618	1	62,628,536	intronic						1E−31	[5]
ADGRL2		rs367881	1	81,883,032	intronic						2E−6	[8]
			1	109,274,968	3'-UTR	c.*919G>T		association			3E−11	[9]
			1	109,275,216	3'-UTR	c.*1167T>C		np			2E−28	[7]
			1	109,275,684	3'-UTR	c.*1635G>T		np			2E−17	[3]
			1	109,279,544	intergenic						2E−11; 2E−19; 2E−31; 4E−289; 7E−9	[5][9]
			1	109,817,590	intergenic						2E−21	[10]
PRPF3		rs56047090	1	150,343,248	intronic						5E−7	[8]
ANXA9		rs267733	1	150,986,360	missense	c.497A>G	p.Asp166Gly	np	probably damaging	tolerated	4E−8	[5]

Mapped gene[a]	Region	SNPid[b]	Chr	Position[c]	Type[d]	cDNA	Protein	ClinVar[e]	Polyphen[f]	Sift[g]	p-value	References
MTARC1	*MTAC1*	rs2642438	1	220,796,686	missense	c.493A>G	p.Thr165Ala	np	benign	tolerated	1E−19	[5]
LINC01132, LINC00184	*IRF2BP2*	rs2587534	1	234,713,592	intergenic						2E−23	[5]
LINC00184 - LINC01132		rs553427	1	234,717,013	intergenic						1E−9	[7]
HS1BP3	*APOB*	rs75352129	2	20,648,432	intronic						6E−59	[7]
LDAH - APOB		rs10172650	2	20,982,585	intergenic						1E−12; 4E−14	[5]
APOB		rs57825321	2	21,024,193	intronic						4E−30	[7]
APOB		rs13306194	2	21,029,662	missense	c.1594C>T	p.Arg532Trp	np	probably damaging	deleterious	1E−12	[3]
APOB		rs1367117	2	21,041,028	missense	c.293C>T	p.Thr98Ile	benign/likely benign	benign	deleterious	2E−10; 2E−179	[5][7]
APOB - TDRD15		rs312949	2	21,111,411	intergenic						3E−6	[2]
TDRD15		chr2:21242731	2	21,242,731	intergenic						1E−18	[10]
TRD15 - NUTF2P8		rs12469758	2	21,310,235	intergenic						5E−53	[7]
VN1R18P		rs77348447	2	48,003,689	intronic						5E−6	[8]
FSHR, CTBP2P5		rs10490120	2	48,916,690	intergenic						1E−6	[8]
EHBP1		rs2710642	2	62,922,422	intronic						2E−10	[5]
Y_RNA, LINC01101		rs2030746	2	120,551,912	intergenic						8E−9	[5]
FN1		rs1250229	2	215,439,661	intergenic						2E−9	[5]
UGT1A8, UGT1A9, UGT1A4, UGT1A7, UGT1A10, UGT1A6, UGT1A5, UGT1A1, UGT1A3		rs11563251	2	233,770,738	intronic						4E−8	[5]
CMTM6		rs3773777	3	32,496,755	intronic						7E−8	[5]
WDR5B-DT, KPNA1		rs3762637	3	122,426,477	intronic						3E−8	[5]
DNAJC13		rs17345563	3	132,490,359	intronic						1E−9	[5]
TNK2		rs75454932 (rs141507877)	3	195,894,179	intronic						3E−6	[8]
RGS12	*LRPAP1*	rs6818397	4	3,433,158	intronic						3E−9	[5]
HEXB	*HMGCR*	rs186072633	5	74,648,603	intronic						6E−15	[10]
HMGCR, ANKRD31		rs6871667	5	75,308,917	intergenic						7E−13	[2]
HMGCR		rs10045497	5	75,340,659	intronic						1E−12	[1]
HMGCR		rs3846661	5	75,343,353	intronic						5E−31	[7]

Mapped gene[a]	Region	SNPid[b]	Chr	Position[c]	Type[d]	cDNA	Protein	ClinVar[e]	Polyphen[f]	Sift[g]	p-value	References
			5	75,352,778	intronic	c.1368+1176A>T		association			1E−6; 1E−9	[9]
			5	75,360,714	3'-UTR	c.*372T>C		np			1E−21; 3E−95	[5]
			5	75,513,055	intronic						1E−16	[5]
CSNK1G3		rs4530754	5	123,519,722	intronic						3E−12	[5]
HAVCR1, TIMD4		rs6882076	5	156,963,286	intergenic						1E−33; 1E−8	[5][7]
			6	16,108,932	intergenic						8E−17	[5]
HLA-C, USP8P1		rs9357121	6	31,272,702	intergenic						3E−10	[2]
HLA-DRA		rs3177928	6	32,444,658	3'-UTR	c.*18G>A		np			5E−17	[5]
TRAM2-AS1		rs2239620	6	52,587,787	intergenic						2E−8	[5]
FRK		rs6909746	6	116,031,587	intronic						2E−9	[5]
HBS1L		rs7775698	6	135,097,497	intronic						2E−8	[5]
HBS1L		rs7776054	6	135,097,778	intronic						4E−8	[5]
H3P28		rs4870470	6	156,593,851	intergenic						2E−6	[8]
DNAH11		rs12670798	7	21,567,734	intronic						2E−12	[5]
MIR148A		rs28537499	7	25,975,772	intergenic						2E−10	[7]
			7	44,539,581	synonymous	c.816C>G	p.Leu272=	benign		tolerated	2E−14	[5]
TBL2		rs17145738	7	73,568,544	3'-UTR	c.*1963G>A					5E−9	[7]
RN7SL265P, ABHD11-AS1		rs112959129	7	73,733,662	intergenic						1E−6	[8]
			8	9,325,848	intergenic						1E−22	[5]
TRMT112P7, RP1		rs10102164	8	54,509,054	intergenic						8E−11	[5]
UBXN2B	*CYP7A1*	rs13277801	8	58,440,975	intronic						4E−19	[5]
CYP7A1, UBXN2B		rs75214121	8	58,485,717	intergenic						9E−9	[7]
TRIB1	*TRIB1*	rs2001846	8	125,466,208	intergenic						6E−9	[7]
TRIB1		rs17321515	8	125,474,167	intergenic						8E−7	[1]
TRIB1		rs2980869	8	125,476,008	intergenic						3E−6; 4E−9	[5]
TRIB1		rs2954029	8	125,478,730	intergenic						2E−56	[5]

Mapped gene[a]	Region	SNPid[b]	Chr	Position[c]	Type[d]	cDNA	Protein	ClinVar[e]	Polyphen[f]	Sift[g]	p-value	References
COL22A1 - KCNK9		rs4588831	8	139,337,989	intergenic						5E−6	[8]
PLEC		rs7832643	8	143,948,489	intronic						2E−17	[5]
			9	2,640,759	intronic						5E−10	[5]
ZNF618		rs59508358 (rs377752731)	9	113,833,966	intergenic						9E−6	[8]
ABO		rs9411378	9	133,270,015	intronic						1E−19	[7]
ABO		rs507666	9	133,273,983	intronic	c.28+1179T>C		association			2E−11	[1]
ABO, Y_RNA		rs579459	9	133,278,724	intergenic						2E−51; 2E−9; 6E−13; 7E−13	[2][5]
TMEM250 - LHX3		chr9:136131651	9	136,131,651	intergenic						5E−11	[10]
OIT3		rs41280378	10	72,932,888	3'-UTR	c.*364T>G		np			9E−15	[7]
GPAM		rs1129555	10	112,150,963	3'-UTR	c.*2587T>C		np			1E−15	[5]
MYRF, TMEM258	*FADS1*	rs174533	11	61,781,553	intronic						1E−9; 5E−7	[5]
FADS2		rs1535	11	61,830,500	intronic						5E−45	[5]
ZPR1	*APOA1-C3-A4-A5*	rs964184	11	116,778,201	3'-UTR	c.*724C>G		np			3E−18	[5]
ZPR1		rs113932726	11	116,779,922	intronic						2E−8	[7]
GSEC, DCPS	*ST3GAL4*	rs2401	11	126,341,832	intronic						5E−8	[5]
GSEC, DCPS		rs17135399	11	126,348,646	intronic						2E−22	[5]
NR1H4		rs75061399	12	100,548,299	intronic						5E−8	[7]
CUX2		rs79105258	12	111,280,427	intronic						3E−20	[7]
RPH3A		rs7315593	12	112,840,165	intronic						8E−9	[7]
			12	120,978,847	missense	c.79A>C	p.Ile27Leu	benign	benign	tolerated	2E−22	[5]
BRCA2		rs1799955	13	32,355,095	synonymous	c.7242A>G	p.Ser2414=	benign		tolerated	3E−8; 3E−9	[5]
BRCA2		rs4942486	13	32,379,251	intronic	c.8755-66T>C		benign			5E−14	[5]
			14	24,414,681	missense	c.2932G>A	p.Ala978Thr	np	benign	tolerated	2E−14	[5]
OTX2-AS1		rs57618243	14	56,984,146	intergenic						7E−6	[8]
			15	58,400,482	intronic						2E−8	[7]

Mapped gene[a]	Region	SNPid[b]	Chr	Position[c]	Type[d]	cDNA	Protein	ClinVar[e]	Polyphen[f]	Sift[g]	p-value	References
CASC22, LINC02180		rs12925859	16	52,194,027	intergenic						4E−6	[8]
HERPUD1, CETP	*CETP*	rs247616	16	56,955,678	intergenic						1E−33	[5]
PKD1L3		rs7185272	16	71,979,898	missense	c.1286C>G	p.Thr429Ser	np	benign	tolerated	5E−8	[3]
PKD1L3		rs7192750	16	71,980,883	intronic						1E−14	[5]
PKD1L3		rs8051431	16	71,981,352	intronic						4E−15	[5]
PKD1L3		rs12927205	16	71,991,178	intronic						8E−7	[2]
TXNL4B		rs77303550	16	72,045,758	intronic						1E−8; 7E−16	[7][9]
HPR, TXNL4B		rs2000999	16	72,074,194	intronic						2E−6; 5E−45	[5]
DLG4		rs314253	17	7,188,331	3'-UTR	c.*2377A>G		np			4E−10	[5]
PFAS		rs4791641	17	8,257,831	missense	c.1100C>T	p.Pro367Leu	np	benign	tolerated	1E−7	[5]
TNFAIP1		rs3093679	17	28,337,189	intronic						2E−9	[7]
			19	11,106,639	missense	c.769C>T	p.Arg257Trp	np	probably damaging	deleterious	3E−8	[3]
			19	11,118,542	intronic						5E−9	[5]
			19	11,131,631	3'-UTR	c.*315G>C		benign/likely benign			3E−15; 4E−40	[5][7]
			19	11,139,463	intronic						1E−24	[2]
			19	11,143,211	intronic						3E−9	[9]
			19	11,145,500	3'-UTR	c.*1683G>A		np			7E−6	[1]
ANGPTL8		rs2278426	19	11,239,812	missense	c.175C>T	p.Arg59Trp	np	possibly damaging	deleterious	2E−9	[7]
SUGP1	*NCAN - TM6SF2*	rs10401969	19	19,296,909	intronic						6E−51	[5]
NECTIN2	*APOE-C1-C2-C4*	rs406456	19	44,879,460	intronic						5E−6	[8]
TOMM40		rs1160985	19	44,900,155	intronic						3E−6; 4E−13	[1][4]
TOMM40 - APOE		rs769446	19	44,905,371	intergenic						3E−322	[7]
APOE		rs429358	19	44,908,684	missense	c.388T>C	p.Cys130Arg	conflicting	benign	tolerated	4E−71	[7]
APOE		rs7412	19	44,908,822	missense	c.526C>T	p.Arg176Cys	drug response	probably damaging	deleterious	2E−286; 7E−15	[5][6]
APOE - APOC1		rs1065853	19	44,909,976	intergenic						3E−54	[9]

Mapped gene[a]	Region	SNPid[b]	Chr	Position[c]	Type[d]	cDNA	Protein	ClinVar[e]	Polyphen[f]	Sift[g]	p-value	References
			19	44,912,383	intergenic						1E−129; 1E−13; 4E−59	[5][6]
			19	45,412,079	intronic						9E−113	[10]
LINC01723		rs364585	20	12,982,070	intergenic						4E−11	[5]
RNU6-192P		rs2328223	20	17,865,277	intergenic						2E−10	[5]
TOP1		rs6065311	20	41,095,698	intronic						2E−34; 3E−6	[5]
ZHX3		rs56668103	20	41,197,439	intronic						7E−9	[7]
MTMR3		rs5763662	22	29,982,714	intronic						3E−9	[5]

[a.]Gene affected by the variant, or the closest genes in case of intergenic variants. [b.]Identification in the dbSNPs database (www.ncbi.nlm.nih.gov/snp). [c.]Positions are relative to genome assembly GRCh38. [d.]Type of mutation. [e.]Classification in the ClinVar database (www.ncbi.nlm.nih.gov/clinvar) of variants affecting coding regions: no interp., no interpretation; np, not present. [f.]Prediction of the functional effect using PolyPhen (genetics.bwh.harvard.edu/pph2/). [g.]Prediction of the functional effect using Sift (sift.bii.a-star.edu.sg/).

Supplementary references for East-Asian populations

1. Zhou, L.; He, M.; Mo, Z.; Wu, C.; Yang, H.; Yu, D.; Yang, X.; Zhang, X.; Wang, Y.; Sun, J., et al. A genome wide association study identifies common variants associated with lipid levels in the Chinese population. *PLoS One* **2013**, *8*, e82420, doi:10.1371/journal.pone.0082420.
2. Lu, X.; Huang, J.; Mo, Z.; He, J.; Wang, L.; Yang, X.; Tan, A.; Chen, S.; Chen, J.; Gu, C.C., et al. Genetic Susceptibility to Lipid Levels and Lipid Change Over Time and Risk of Incident Hyperlipidemia in Chinese Populations. *Circ Cardiovasc Genet* **2016**, *9*, 37-44, doi:10.1161/CIRCGENETICS.115.001096.
3. Tang, C.S.; Zhang, H.; Cheung, C.Y.; Xu, M.; Ho, J.C.; Zhou, W.; Cherny, S.S.; Zhang, Y.; Holmen, O.; Au, K.W., et al. Exome-wide association analysis reveals novel coding sequence variants associated with lipid traits in Chinese. *Nat Commun* **2015**, *6*, 10206, doi:10.1038/ncomms10206.
4. Kurano, M.; Tsukamoto, K.; Kamitsuji, S.; Kamatani, N.; Hara, M.; Ishikawa, T.; Kim, B.J.; Moon, S.; Jin Kim, Y.; Teramoto, T. Genome-wide association study of serum lipids confirms previously reported associations as well as new associations of common SNPs within PCSK7 gene with triglyceride. *J Hum Genet* **2016**, *61*, 427-433, doi:10.1038/jhg.2015.170.
5. Spracklen, C.N.; Chen, P.; Kim, Y.J.; Wang, X.; Cai, H.; Li, S.; Long, J.; Wu, Y.; Wang, Y.X.; Takeuchi, F., et al. Association analyses of East Asian individuals and trans-ancestry analyses with European individuals reveal new loci associated with cholesterol and triglyceride levels. *Hum Mol Genet* **2017**, *26*, 1770-1784, doi:10.1093/hmg/ddx062.
6. Zhu, Y.; Zhang, D.; Zhou, D.; Li, Z.; Li, Z.; Fang, L.; Yang, M.; Shan, Z.; Li, H.; Chen, J., et al. Susceptibility loci for metabolic syndrome and metabolic components identified in Han Chinese: a multi-stage genome-wide association study. *J Cell Mol Med* **2017**, *21*, 1106-1116, doi:10.1111/jcmm.13042.
7. Kanai, M.; Akiyama, M.; Takahashi, A.; Matoba, N.; Momozawa, Y.; Ikeda, M.; Iwata, N.; Ikegawa, S.; Hirata, M.; Matsuda, K., et al. Genetic analysis of quantitative traits in the Japanese population links cell types to complex human diseases. *Nat Genet* **2018**, *50*, 390-400, doi:10.1038/s41588-018-0047-6.
8. Liu, H.; Wang, W.; Zhang, C.; Xu, C.; Duan, H.; Tian, X.; Zhang, D. Heritability and Genome-Wide Association Study of Plasma Cholesterol in Chinese Adult Twins. *Front Endocrinol (Lausanne)* **2018**, *9*, 677, doi:10.3389/fendo.2018.00677.
9. Moon, S.; Kim, Y.J.; Han, S.; Hwang, M.Y.; Shin, D.M.; Park, M.Y.; Lu, Y.; Yoon, K.; Jang, H.M.; Kim, Y.K., et al. The Korea Biobank Array: Design and Identification of Coding Variants Associated with Blood Biochemical Traits. *Sci Rep* **2019**, *9*, 1382, doi:10.1038/s41598-018-37832-9.
10. Han, S.; Hwang, M.Y.; Yoon, K.; Kim, Y.K.; Kim, Y.J.; Kim, B.J.; Moon, S. Exome chip-driven association study of lipidemia in >14,000 Koreans and evaluation of genetic effect on identified variants between different ethnic groups. *Genet Epidemiol* **2019**, *43*, 617-628, doi:10.1002/gepi.22208.

Table S1c: SNVs associated with low-density lipoprotein cholesterol in Afro-Americans or African ancestry populations.

Mapped gene[a]	Region	SNPid[b]	Chr	Position[c]	Type[d]	cDNA	Protein	ClinVar[e]	Polyphen[f]	Sift[g]	p-value	References
PCSK9	*PCSK9*	rs28362286	1	55,063,542	nonsense	c.2037C>A	p.Cys679Ter	conflicting			2E−42	[3]
CELSR2	*CELSR2 - SORT1*	rs12740374	1	109,274,968	3'-UTR	c.*919G>T		association			2E−26; 9E−29	[1][3]
TARBP1 - IRF2BP2	*IRF2BP2*	rs744487	1	234,590,266	intergenic						5E−6	[1]
			2	20,901,018	intergenic						1E−7	[1]
			2	21,191,270	intergenic						3E−9	[1]
			2	22,599,287	intergenic						4E−7	[1]
DTNB		rs11684202	2	25,664,689	intronic						6E−6	[1]
MYO3B - SP5		rs2080401	2	170,684,313	intergenic						7E−6	[1]
TNIK		rs11920719	3	171,116,770	intronic						4E−6	[1]
SGCD		chr5:156378584	5	156,378,584	intronic						5E−9	[3]
RNF130		rs13161895	5	180,044,201	intronic						4E−7	[1]
PDGFD		rs10895547	11	103,937,424	intronic						3E−6	[1]
PLEKHO2		rs12595292	15	64,861,491	synonymous	c.399G>A	p.Lys133=	np			9E−6	[1]
LITAF		rs7203193	16	11,547,324	intergenic						3E−6	[1]
ZFHX3		rs16971384	16	72,897,186	intronic						5E−6	[1]
DNM2	*LDLR*	rs11671653	19	10,727,810	intronic						9E−7	[1]
SMARCA4		rs11669133	19	10,981,463	intronic						1E−8	[1]
SMARCA4 - LDLR		rs12151108	19	11,086,585	intergenic						2E−32	[3]
LDLR		rs6511720	19	11,091,630	intronic			benign			7E−8	[1]
APOE	*APOE-C1-C2-C4*	rs7412	19	44,908,822	missense	c.526C>T	p.Arg176Cys	drug response	probably damaging	deleterious	1E−189; 2E−9	[2][3]
LOC105373347		rs5904726	X	147,241,105	intronic						9E−6	[1]

[a] Gene affected by the variant, or the closest genes in case of intergenic variants. [b] Identification in the dbSNPs database (www.ncbi.nlm.nih.gov/snp). [c] Positions are relative to genome assembly GRCh38. [d] Type of mutation. [e] Classification in the ClinVar database (www.ncbi.nlm.nih.gov/clinvar) of variants affecting coding regions: no interp., no interpretation; np, not present. [f] Prediction of the functional effect using PolyPhen (genetics.bwh.harvard.edu/pph2/). [g] Prediction of the functional effect using Sift (sift.bii.a-star.edu.sg/).

Table S1d: SNVs associated with low-density lipoprotein cholesterol in Hispanic or Native-American ancestry populations.

Mapped gene[a]	Region	SNPid[b]	Chr	Position[c]	Type[d]	cDNA	Protein	ClinVar[e]	Polyphen[f]	Sift[g]	p-value	References
CELSR2	*CELSR2 - SORT1*	rs12740374	1	109,274,968	3'-UTR	c.*919G>T		association			1E−36	[2]
CELSR2		rs660240	1	109,275,216	3'-UTR	c.*1167T>C		np			1E−28	[1]
APOB	*APOB*	rs13392272	2	20,994,618	intergenic						2E−12	[1]
TDRD15, APOB		rs7575840	2	21,050,618	intergenic						4E−18	[2]
APOB, TDRD15		rs562338	2	21,065,449	intergenic						3E−19	[2]
ABCG8	*ABCG5/G8*	rs4245791	2	43,847,292	intronic						5E−8	[2]
HMGCR	*HMGCR*	rs6882842	5	75,356,084	intronic						4E−6	[2]
ZNF619P1		rs6944635	7	46,132,286	intergenic						3E−6	[2]
			11	61,781,553	intronic						6E−7	[2]
DHODH		rs8062895	16	72,014,733	intronic						3E−6	[2]
PFAS		rs4791641	17	8,257,831	missense	c.1100C>T	p.Pro367Leu	np	benign	tolerated	1E−8	[1]
LDLR	*LDLR*	rs6511720	19	11,091,630	intronic	c.67+2015G>T		benign			2E−18	[2]
NCAN	*NCAN - TM6SF2*	rs2238675	19	19,225,799	intronic						2E−8	[1]
NECTIN2	*APOE-C1-C2-C4*	rs7254892	19	44,886,339	intronic						2E−38	[2]

[a.] Gene affected by the variant, or the closest genes in case of intergenic variants. [b.] Identification in the dbSNPs database (www.ncbi.nlm.nih.gov/snp). [c.] Positions are relative to genome assembly GRCh38. [d.] Type of mutation. [e.] Classification in the ClinVar database (www.ncbi.nlm.nih.gov/clinvar) of variants affecting coding regions: no interp., no interpretation; np, not present. [f.] Prediction of the functional effect using PolyPhen (genetics.bwh.harvard.edu/pph2/). [g.] Prediction of the functional effect using Sift (sift.bii.a-star.edu.sg/)

Table S1e: SNVs associated with low-density lipoprotein cholesterol in Middle-East populations.

Mapped gene[a]	Region	SNPid[b]	Chr	Position[c]	Type[d]	cDNA	Protein	ClinVar[e]	Polyphen[f]	Sift[g]	p-value	References
CELSR2	*CELSR2 - SORT1*	rs7528419	1	109,274,570	3'-UTR variant	c.*521A>G		np			5E−6	[1]
TDRD15	*APOB*	rs70939068 (rs142138117)	2	21,124,818	intronic variant						7E−8	[1]
LOC105374317		rs10183198	2	21,248,115	intronic variant						5E−7	[1]
INTS10, LPL	*LPL*	rs10635970	8	19,887,529	A repetition						2E−8	[1]
RPL30P9, LPL		rs7016880	8	20,019,235	intergenic						5E−6	[1]
C8orf37-AS1, LINC01298		rs62522646	8	95,211,565	intronic variant						1E−7	[1]
			19	11,165,580	3'-UTR variant	c.*978A>G		np			9E−8	[1]

[a]Gene affected by the variant, or the closest genes in case of intergenic variants. [b]Identification in the dbSNPs database (www.ncbi.nlm.nih.gov/snp). [c]Positions are relative to genome assembly GRCh38. [d]Type of mutation. [e]Classification in the ClinVar database (www.ncbi.nlm.nih.gov/clinvar) of variants affecting coding regions: no interp., no interpretation; np, not present. [f]Prediction of the functional effect using PolyPhen (genetics.bwh.harvard.edu/pph2/). [g]Prediction of the functional effect using Sift (sift.bii.a-star.edu.sg

Table S1f: SNVs associated with low-density lipoprotein cholesterol in Oceanian populations.

Mapped gene[a]	Region	SNPid[b]	Chr	Position[c]	Type[d]	cDNA	Protein	ClinVar[e]	Polyphen[f]	Sift[g]	p-value	References
APOB - TDRD15	*APOB*	rs754523	2	21,088,819	intergenic						6E−6	[2]
ANKRD31, HMGCR	*HMGCR*	rs7703051	5	75,329,662	intergenic						1E−8	[1]
TOMM40	*APOE-C1-C2-C4*	rs1160985	19	44,900,155	intronic						2E−27	[2]
APOC1		rs4420638	19	44,919,689	downstream	c.*459A>G		no interp.			2E−7	[1]

[a]Gene affected by the variant, or the closest genes in case of intergenic variants. [b]Identification in the dbSNPs database (www.ncbi.nlm.nih.gov/snp). [c]Positions are relative to genome assembly GRCh38. [d]Type of mutation. [e]Classification in the ClinVar database (www.ncbi.nlm.nih.gov/clinvar) of variants affecting coding regions: no interp., no interpretation; np, not present. [f]Prediction of the functional effect using PolyPhen (genetics.bwh.harvard.edu/pph2/). [g]Prediction of the functional effect using Sift (sift.bii.a-star.edu.sg/).

Supplementary references for Afro-American or African ancestry populations

1. Lettre, G.; Palmer, C.D.; Young, T.; Ejebe, K.G.; Allayee, H.; Benjamin, E.J.; Bennett, F.; Bowden, D.W.; Chakravarti, A.; Dreisbach, A., et al. Genome-wide association study of coronary heart disease and its risk factors in 8,090 African Americans: the NHLBI CARe Project. *PLoS Genet* **2011**, *7*, e1001300, doi:10.1371/journal.pgen.1001300.
2. Rasmussen-Torvik, L.J.; Pacheco, J.A.; Wilke, R.A.; Thompson, W.K.; Ritchie, M.D.; Kho, A.N.; Muthalagu, A.; Hayes, M.G.; Armstrong, L.L.; Scheftner, D.A., et al. High density GWAS for LDL cholesterol in African Americans using electronic medical records reveals a strong protective variant in APOE. *Clin Transl Sci* **2012**, *5*, 394-399, doi:10.1111/j.1752-8062.2012.00446.x.
3. Gurdasani, D.; Carstensen, T.; Fatumo, S.; Chen, G.; Franklin, C.S.; Prado-Martinez, J.; Bouman, H.; Abascal, F.; Haber, M.; Tachmazidou, I., et al. Uganda Genome Resource Enables Insights into Population History and Genomic Discovery in Africa. *Cell* **2019**, *179*, 984-1002 e1036, doi:10.1016/j.cell.2019.10.004.

Supplementary references for Native American or Hispanic populations

1. Below, J.E.; Parra, E.J.; Gamazon, E.R.; Torres, J.; Krithika, S.; Candille, S.; Lu, Y.; Manichakul, A.; Peralta-Romero, J.; Duan, Q., et al. Meta-analysis of lipid-traits in Hispanics identifies novel loci, population-specific effects, and tissue-specific enrichment of eQTLs. *Sci Rep* **2016**, *6*, 19429, doi:10.1038/srep19429.
2. Andaleon, A.; Mogil, L.S.; Wheeler, H.E. Genetically regulated gene expression underlies lipid traits in Hispanic cohorts. *PLoS One* **2019**, *14*, e0220827, doi:10.1371/journal.pone.0220827.

Supplementary references for Middle-East populations.

1. Hebbar, P.; Abubaker, J.A.; Abu-Farha, M.; Alsmadi, O.; Elkum, N.; Alkayal, F.; John, S.E.; Channanath, A.; Iqbal, R.; Pitkaniemi, J., et al. Genome-wide landscape establishes novel association signals for metabolic traits in the Arab population. *Hum Genet* **2021**, *140*, 505-528, doi:10.1007/s00439-020-02222-7.

Supplementary references for Oceanian populations.

1. Burkhardt, R.; Kenny, E.E.; Lowe, J.K.; Birkeland, A.; Josowitz, R.; Noel, M.; Salit, J.; Maller, J.B.; Pe'er, I.; Daly, M.J., et al. Common SNPs in HMGCR in micronesians and whites associated with LDL-cholesterol levels affect alternative splicing of exon13. *Arterioscler Thromb Vasc Biol* **2008**, *28*, 2078-2084, doi:10.1161/ATVBAHA.108.172288.
2. Carlson, J.C.; Weeks, D.E.; Hawley, N.L.; Sun, G.; Cheng, H.; Naseri, T.; Reupena, M.S.; Tuitele, J.; Deka, R.; McGarvey, S.T., et al. Genome-wide association studies in Samoans give insight into the genetic architecture of fasting serum lipid levels. *J Hum Genet* **2020**, *66*, 111-121, doi:10.1038/s10038-020-0816-9.
